# Exercise-therapy and education for individuals one year after anterior cruciate ligament reconstruction: a pilot randomised controlled trial

**DOI:** 10.1186/s12891-020-03919-6

**Published:** 2021-01-11

**Authors:** Brooke E. Patterson, Christian J. Barton, Adam G. Culvenor, Randall L. Cooper, Kay M. Crossley

**Affiliations:** La Trobe Sport & Exercise Medicine Research Centre, School of Allied Health, Humans Services and Sport, La Trobe University, Bundoora, 3086 Australia

**Keywords:** Anterior cruciate ligament, Rehabilitation, Physiotherapy

## Abstract

**Background:**

Guided rehabilitation beyond 6-months is rare following anterior cruciate ligament reconstruction (ACLR), despite high prevalence of unacceptable symptoms and quality of life (QoL). Our primary aim was to determine the feasibility of a randomised controlled trial (RCT) evaluating a physiotherapist-guided intervention for individuals 1-year post-ACLR with persistent symptoms. Our secondary aim was to determine if a worthwhile treatment effect could be observed for the lower-limb focussed intervention (compared to the trunk-focussed intervention), for improvement in knee-related QoL, symptoms, and function.

**Design:**

Participant- and assessor-blinded, pilot feasibility RCT.

**Methods:**

Participant eligibility criteria: i) 12–15 months post-ACLR; ii) < 87.5/100 on the Knee injury and Osteoarthritis Outcome Score (KOOS) QoL subscale; and either a one-leg rise test < 22 repetitions, single-hop < 90% limb symmetry; or Anterior Knee Pain Scale < 87/100. Participants were randomised to lower-limb or trunk-focussed focussed exercise and education. Both interventions involved 8 face-to-face physiotherapy sessions over 16-weeks. Feasibility was assessed by eligibility rate (> 1 in 3 screened), recruitment rate (> 4 participants/month), retention (< 20% drop-out), physiotherapy attendance and unsupervised exercise adherence (> 80%). Between-group differences for knee-related QoL (KOOS-QoL, ACL-QoL), symptoms (KOOS-Pain, KOOS-Symptoms), and function (KOOS-Sport, functional performance tests) were used to verify that the worthwhile effect (greater than the minimal detectable change for each measure) was contained within the 95% confidence interval.

**Results:**

47% of those screened were eligible, and 27 participants (3 participants/month; 48% men, 34±12 years) were randomised. Two did not commence treatment, and two were lost to follow-up (16% drop-out). Physiotherapy attendance was > 80% for both groups but reported adherence to unsupervised exercise was low (< 55%). Both interventions had potentially worthwhile effects for KOOS-QoL and ACL-QoL, while the lower-limb focussed intervention had potentially greater effects for KOOS-Sport, KOOS-Pain, and functional performance.

**Conclusions:**

A larger-scale RCT is warranted. All feasibility criteria were met, or reasonable recommendations could be made to achieve the criteria in future trials. Strategies to increase recruitment rate and exercise adherence are required. The potential worthwhile effects for knee-related QoL, symptoms, and function indicates a fully-powered RCT may detect a clinically meaningful effect.

**Trial registration:**

Prospectively registered (ACTRN12616000564459).

**Supplementary Information:**

The online version contains supplementary material available at 10.1186/s12891-020-03919-6.

## Background

Following anterior cruciate ligament reconstruction (ACLR), clinical practice guidelines recommend post-operative rehabilitation to continue for at least 9 to 12 months, or until achievement of sport-specific strength, functional and psychological criteria [72]. Yet, many patients have symptoms, muscle weakness and functional deficits that persist beyond 1-year post-ACLR [13, 55, 66, 79], which may increase the risk of re-injury, symptomatic post-traumatic osteoarthritis (OA), and worse knee-related quality of life (QoL) [17, 18, 25, 26, 33, 53]. Yet, there are no clinical trials to suggest exercise and education beyond the typical rehabilitation period is feasible or beneficial for those who have not achieved an acceptable outcome within the first post-operative year.

Approximately 50% of individuals report unacceptable knee symptoms and QoL 1 to 2 years after ACLR [39, 55]. Minimal improvement occurs beyond 1 to 2 years [55, 65], and symptoms and QoL remain worse than their uninjured peers in the longer-term (> 5 years) [27, 55]. Persistent symptoms at 1-year post-ACLR often co-exist with impairments in physical strength and function, and loss of knee confidence [16, 35]. Strength and functional performance impairments are typically defined as a difference in performance greater than 10% between the ACLR and contralateral limb. Persistent symptoms and functional deficits at 1-year post-ACLR increase the risk of developing short-term (< 5 years) and longer-term (5 to 10 years) symptoms, impaired knee-related QoL and OA [15, 25, 53]. Therefore, the one-year post-operative milestone provides an ideal window to identify “at risk individuals” with persistent symptoms, who have ceased supervised rehabilitation and for interventions to be implemented. Physiotherapist-guided exercise-therapy and education to address persistent physical impairments and symptoms, may be important to the secondary prevention of re-injury, post-traumatic OA, and poor QoL in young adults post-ACLR [12, 76].

The primary aim of this pilot study was to determine the feasibility of a randomised controlled trial (RCT) evaluating a physiotherapist-guided exercise-therapy intervention for individuals with persistent symptoms 1-year post-ACLR. Our secondary aim was to determine if a worthwhile treatment effect was observed for the lower-limb focussed intervention (compared to the trunk-focussed intervention), for improvement in knee-related QoL, symptoms, and function.

## Methods

### Study design

This double-blind (assessor and participant), parallel-arm, pilot feasibility RCT was conducted in accordance with the National Health and Medical Research Council ethical guidelines [51], and reporting adheres to the Consolidated Standards of Reporting Trials (CONSORT) statement for pilot and feasibility studies [23] (Additional file 1). Ethical approval was gained from the La Trobe University Human Ethics Committee (HEC 16–077). The trial was prospectively registered through the Australia and New Zealand Clinical Trials Registry (ACTRN12616000564459).

### Setting

All assessments and treatments were conducted at two private physiotherapy clinics in Australia, located in Hobart or Melbourne.

### Participant recruitment and eligibility

Individuals who had undergone a hamstring-tendon autograft ACLR 12 to 15 months prior were recruited from five surgical lists, advertisements at La Trobe University, or via social media (December 2016 to August 2017). Individuals aged 18 to 50 years who were 12 to 15 months post-ACLR were considered eligible if they scored < 87.5/100 on the Knee injury and Osteoarthritis Outcome Score (KOOS) QoL subscale (threshold below which has been defined a symptomatic knee [24]), and met one of the following criteria; a) < 22 repetitions on the one-leg rise test; b) single-hop < 90% limb symmetry index (LSI); or c) < 87/100 on the Anterior Knee Pain Scale (AKPS) [44]. These functional performance thresholds can be associated with worse symptoms and poorer knee-related QoL in the proceeding years [15, 25, 61], and the AKPS threshold can be associated with worse functional performance at 1-year post-ACLR [16]. Exclusion criteria were: i) > 5 years between injury and ACLR; ii) subsequent injury (for which medical treatment was sought) or follow-up surgery to the ACLR knee; iii) another musculoskeletal, neurological, or cardiorespiratory condition influencing daily function; iv) unable to speak or read English; and v) unable to attend eight supervised sessions.

### Deviations from initial trial protocol

Participants were initially deemed ineligible if they had sustained a previous ACL or knee injury to either limb prior to their recent ACLR. After commencing recruitment, it was evident that a previous knee injury was common in those with persistent symptoms, and these individuals have an increased risk of symptomatic post-traumatic OA [78]. The inclusion criteria were adjusted at the start of recruitment to include those with a previous ACL or knee injury. The single-hop performance LSI cut-off for eligibility was changed from 88 to 90%, as recent evidence suggests 90% is the most common criterion used for return-to-sport clearance [9]. Hypothesis testing in a regression model was not performed as initially planned, due to the limitations of significance testing in clinical research [36], and this was not considered appropriate for a feasibility trial. Instead, the between-group differences and 95% confidence intervals (CI) were used to verify that a worthwhile effect was contained within the CI [5]. We defined a potential worthwhile effect as greater than the minimal detectable change (MDC) score for the respective outcome measures where available. While the primary purpose of feasibility was implied throughout the trial registration and included as such in the trial title, we did not list feasibility as a separate outcome in the trial registration. We have maintained our focus on feasibility by including it as the primary aim of this pilot study. Several other exploratory PROs were outlined in the trial registration but were beyond the scope of this evaluation due to the primary aim of feasibility.

### Procedures

Eligible participants underwent a baseline assessment with a blinded assessor (BP) and were randomised into one of two intervention groups. The same blinded assessor completed all follow-up assessments unaware of group allocation. Participant age, sex, body mass index (BMI), injury history, ACLR rehabilitation (i.e. self-reported duration) and surgical details (i.e. self-reported graft type, meniscal procedures), and previous activity level were obtained at baseline. All patient-reported outcomes (PROs) were completed via an online portal (Promptus, DS PRIMA, Melbourne, Australia).

#### Randomisation and blinding

Non-stratified, permuted block randomisation (random blocks of 3 or 6) occurred at a 2:1 (lower-limb focussed: trunk-focussed) ratio. The randomisation sequence was computer-generated using Excel. The administrative staff at the participating physiotherapy clinic revealed the allocation using sequentially numbered, sealed opaque envelopes. The administrative staff were blinded to block size, and entered the group allocation to the participant’s clinical record for the physiotherapist. Participants were blinded to group allocation, to ensure allocation did not influence adherence, other treatment use, or increase the risk of drop-out. The physiotherapists were unable to be blinded to the allocation but were encouraged to deliver both interventions with equal enthusiasm and assertion of exercise value.

#### Treating physiotherapists and treatment fidelity

Treating physiotherapists were experienced (≥5 years treating musculoskeletal patients) in ACLR rehabilitation, and completed a 4-h training session (led by BP) related to delivering both interventions. A manual, outlining the exercise prescription and progressions, manual treatment algorithm, education material, and trial procedures (attendance sheet, clinical notes, adherence monitoring) was provided to each physiotherapist (Additional file 2). Prescribed exercises were entered via Physitrack© smartphone application for participants to access via the participant-facing application PhysiApp© (Physitrack Ltd., London, UK).

### Interventions

Participants were randomised to a lower-limb focussed or trunk-focussed exercise-therapy intervention, which were both delivered in eight face-to-face 30-min physiotherapy sessions over 16-weeks. Both interventions are reported below according to the Template for Intervention Description and Replication (TIDieR) guidelines [38] and the Consensus on Exercise Reporting Template (CERT) [64] (Table 1).

**Table 1:** Summary of intervention delivery and components for both groups

What	Lower-limb focussed intervention	Trunk-focussed intervention
Who provides	Physiotherapists who have all undergone study-specific training	
How	1-to-1 face-to-face sessions to assess and progress unsupervised exercise-therapy program	
Where	*Physiotherapy sessions:* Private clinics in Hobart and Melbourne *Unsupervised exercise-therapy program:* Clinic/public gym, or home	
When & how much	*Physiotherapy 1-to-1 sessions:* 30 min duration, weekly for 4 weeks then every 2 to 3 weeks for 12 weeks *Unsupervised exercise-therapy program:* instructions provided via PhysiApp©, 30 to 45 min duration, minimum 3 sessions per week, unsupervised	
Tailoring	• Standardised lower-limb exercises (i.e. strength, power, balance), functional retraining (e.g. plyometric, agility) and cardiovascular program • Choice of priority exercises^a^ (from the standard set) was individualised • Exercise progression was individualised • Individualised education (e.g. exercise rationale, goal setting) • Passive therapy treatment algorithm if appropriate (e.g. taping)	• Standardised, non-specific trunk strengthening exercises • Progression of exercises was individualised • Optional stretching • Standardised education (e.g. rationale for trunk exercises)
	*Both groups: exercises progressed based on assessment of pre-defined criteria at each session (*i.e.*, pain, swelling, technique) and resistance training principles*	
How well	Attendance at physiotherapy recorded by physiotherapists and clinic Unsupervised exercise program adherence recorded by participants in PhysiApp© smartphone app or paper diaries, and monitored by physiotherapists via Physitrack©	

^a^ Physiotherapists could choose 3 to 4 priority exercises (out of a possible 8), based on the participant’s needs and goals. If necessary, all 8 exercise types were included, but it was not compulsory for all eight to be incorporated

#### Lower-limb focussed exercise-therapy intervention

The lower-limb focussed intervention included standardised (with individualised progression) lower-limb, functional and cardiovascular exercises, and individualised, ACL-specific education (Additional file 2). The protocol was informed by current evidence-based recommendations [72], and developed by the research team, two of whom regularly (weekly) treated patients after ACLR (CB and RC). The lower-limb focussed exercise-therapy program targeted typical strength and functional impairments [79], and altered movement patterns [79] during sport-specific tasks related to ACL injury mechanisms (i.e., landing, and cutting). The eight areas in the exercise-program were: 1) movement retraining (e.g. landing); 2) lower-limb strength (e.g. squats); 3) balance (e.g. perturbation exercises); 4) hip-abductor strength; 5) calf strength; 6) trunk strength; 7) hip-extensor and knee-flexor strength; and 8) cardiovascular exercise (e.g. cycling, running, graded sport-specific activities). Each of the eight exercises had three or more phases of difficulty for individualised progression (Additional file 2). Physiotherapists were provided with a summary of the participant’s injury history, goals, 3 to 4 priority exercises, and suggested starting phases based on baseline assessment. Physiotherapists could add target exercises based on participant need, but it was not compulsory for all eight exercises to be incorporated. Exercise progression was based on: i) good technique; ii) minimal irritability (i.e. < 2/10 pain during/after and no swelling); iii) resistance training principles related to muscular strength and power [1]; and iv) participant-specific goals and feedback. Strength exercises were prescribed in 3 sets of 12 repetitions (each repetition performed as 2 s concentric, 1 s isometric, 2 s eccentric), and could be progressed to a power dosage prescribed in 3 to 5 sets of 5 to 10 repetitions (< 1 s concentric, 0 isometric, 2 s eccentric) [1]. Treating physiotherapists were encouraged to use the face-to-face sessions to check exercise technique, and adjust loads so that participants were reaching fatigue (i.e. they could not physically perform > 2 more repetitions) after their prescribed dosage [1]. Thirty-minutes was considered an appropriate appointment duration to supervise at least 1 set of prescribed exercises (the other 2 sets could be completed unsupervised in the clinic gym), and provide education.

#### Trunk-focussed exercise-therapy (control) intervention

An active control intervention was chosen to ensure that both treatment groups received equal exposure to physiotherapy [37]. The trunk-focussed intervention was considered the active control, and included standardised (with individualised progression) trunk strengthening exercises, stretching and education. Physiotherapists could choose a minimum of three trunk strengthening exercises (from a maximum of five options), and each exercise had three or more phases of difficulty (Additional file 2). Exercises were prescribed according to resistance training principles; typically prescribed in 3 sets of 60 s (isometric), and progressed to achieve adequate fatigue (i.e., could not physically perform > 5 more seconds) [1]. Lower-limb and trunk stretching appropriate to the participant, could be prescribed (Additional file 2). The trunk exercises were predominantly isometric, non-weight-bearing, and had minimal lower-limb involvement and thus, were not expected to impact knee-related QoL, symptoms, or function. This was chosen as the control intervention as trunk muscle strength deficits has not been reported following ACLR, nor has addressing trunk strength been investigated to impact knee-related outcomes following ACLR. Trunk exercises were considered to provide a credible intervention to enhance control participant’s blinding to group allocation and minimise drop-outs.

#### Unsupervised exercise-therapy program (both groups)

Participants in both groups were prescribed an unsupervised exercise-therapy program relevant to their allocation, to be completed 3 times per week, at home or in a gym, to optimise likelihood of muscular strength and power improvements [29]. Physiotherapists entered participant’s exercises via the Physitrack© app, for the participant to use PhysiApp© to guide exercises and record adherence on their own smartphone, tablet or computer. Paper diaries of the exercise-therapy programs were provided as required. PhysiApp© included video examples (created specifically for the trial) of correct (and incorrect) technique for each exercise (Additional file 3), and exercise dosage (e.g. number of sets/repetitions, time under tension, external load, rest time) according to resistance training and muscle adaptation guidelines [1, 69]. Co-interventions were discouraged but if participants chose to receive other treatment, they recorded them on an “other treatments calendar”. The trunk-focussed unsupervised program could be completed at home with minimal equipment. When gym equipment was required for the lower-limb focussed unsupervised program, gym access was provided free of charge.

#### Education component (both groups)

Both groups received education, including face-to-face discussion and/or provision of handouts (Additional file 3). Handouts for the lower-limb focussed group covered the following topics: i) surgical information and post-operative expectations; ii) goal setting and return-to-sport criteria; iii) injury prevention; iv) psychosocial influences on recovery; and v) post-traumatic OA risk. The purpose of the education for the lower-limb focussed group was to provide informational support regarding ACL-specific topics and address common knowledge gaps regarding evidence-based rehabilitation [6], and psychosocial support for kinesiophobia, fear of re-injury, confidence, or negative lifestyle modifications (e.g. weight gain) known to influence outcomes [11, 56, 70]. For the trunk-focussed group, physiotherapists could deliver standardised education on the rationale for trunk strengthening (e.g. theoretical influence of lumbo-pelvic stability on lower-limb biomechanics), or provide handouts/ face-to-face discussion on the topics “surgical information and post-operative expectations”, “psychosocial influences on recovery”, and “post-traumatic OA risk” (Additional file 3).

### Primary outcome: feasibility

Feasibility was assessed according to previously published recommendations [47]. Proceeding to a full-scale RCT was deemed feasible if all criteria were met, or reasonable amendments could be made to achieve these criteria in future trials [3].

***Recruitment, adherence and retention*** was evaluated by: i) recruitment rate (criterion: 4 participants per month); ii) proportion of eligible participants who were willing to enrol (criterion: > 80%); iii) physiotherapy attendance rate (criterion: > 80%); iv) adherence to unsupervised exercise-therapy program (criterion: > 80%); and v) proportion of drop-outs (criterion: < 20%).

***Acceptability of the study protocol*** was assessed via the appropriateness of the inclusion criteria (criterion: at least 1 in 3 eligible), and acceptability of the intervention content, delivery, adherence monitoring, and barriers or facilitators to adherence. Acceptability was determined via informal interviews conducted with participants and physiotherapists (Additional file 4).

***Adverse events*** (i.e., any injury or illness requiring medical attention as a result of participating in the trial)) were noted by the physiotherapist on a standardised recording sheet (criterion: < 10% of all participants). Pain-level (on a visual analogue scale; 0=no pain, 10=worst possible pain) during the unsupervised exercise-therapy program was entered on PhysiApp© by participants (criterion: each participant mean < 2/10 across all sessions).

***Randomisation integrity*** was determined by contamination between groups (reported by participant or physiotherapist) (criterion: 0% contamination), or knowledge of group allocation by the participants or assessor (criterion: < 10% unblinded).

***Acceptability of the outcome measures*** was determined by the time needed to collect the data, and completeness of the outcome measures at baseline and follow-up (criterion: > 90%).

### Secondary outcomes

#### Patient-reported outcomes

Knee-related QoL was assessed via the KOOS-QoL and ACL-QoL. The KOOS-QoL is one of the five KOOS subscales, and evaluates knee-related QoL [63]. The KOOS-QoL has the highest content validity of all subscales and the greatest responsiveness in young adults following knee injury [10]. The ACL-QoL was designed to assess additional domains (e.g. work-related, social and emotional) of knee-related QoL specific to a young, active ACL-injured population [49]. The KOOS-QoL and ACL-QoL are converted to a total score out of 100 (0=extreme problems, 100=no problems). The KOOS-QoL and ACL-QoL have established content validity (Cronbach’s alpha > 0.76), test-retest reliability (ICC> 0.86), and responsiveness (effect sizes > 0.5) [10, 46]. The MDC is 8–10 points for KOOS-QoL [63], and 12-points for ACL-QoL [46].

The KOOS subscales of pain, symptoms, and sport were assessed, and all combined with the KOOS-QoL, to calculate an overall KOOS_4_ score. The KOOS individual subscales are valid, reliable and responsive following ACL injury [10]. Psychological readiness for return-to-sport (a common goal of ACLR), and fear of re-injury was measured by the ACL Return to Sport Index (ACL-RSI) [74]. The ACL-RSI has established test-retest reliability (ICC=0.89) and responsiveness (MDC=19 points) [45], and validity with higher scores associated with better return-to-sport rates, self-reported symptoms and function [45, 75]. The global rating of change (GROC) on a 7-point Likert scale (“much worse” to “much better”) measured separately for knee pain and knee function; and the change in proportion of patients answering “yes” to the patient acceptable symptom state (PASS) question [39] were evaluated. The GROC has good face validity (Pearson’s r=0.72 to 0.90), test-retest reliability (ICC> 0.90), responsiveness following knee injury (0.5 to 2.7 points on 7-point scale), and construct validity (e.g. correlated with changes in hop tests) [40]. The PASS assists in interpretation of improvement in PROs by evaluating the concept of “feeling good” as opposed to “feeling better” [71]¸ and answering yes to “PASS” corresponds with better KOOS scores after ACL injury [39].

#### Functional performance outcomes

Functional performance outcomes were measured at baseline and follow-up, including the single-hop (maximum distance on one hop forward) [34], side-hop (maximum number of hops over two parallel lines 40 cm apart in 30 s) [34], and one-leg rise test (maximum number repetitions from a standardised height) [68]. We recorded the raw score (e.g. cm hopped) on the ACLR and contralateral limb, and calculated the LSI (score of ACLR knee divided by contralateral knee, multiplied by 100, expressed a percentage). The hop-tests and one-leg rise have high intra-rater reliability (ICC> 0.80) and responsiveness after knee injury [7, 34, 60].

### Data analysis

The sample size of 27 was not formally determined. It was based on previous pilot RCTs evaluating health-professional guided interventions for musculoskeletal conditions [41, 67], and deemed sufficient to assess the feasibility criteria. Participants who completed baseline and follow-up evaluations were included in the analysis, as recommended in the CONSORT guidelines [48]. Feasibility outcomes were reported descriptively. The majority (> 50%) of baseline and follow-up scores, and the change scores for the patient-reported and functional performance outcomes were normally distributed (assessed with Shapiro-Wilk’s test). Therefore, within-group, and between-group differences were reported as mean±SD, and mean and 95% confidence interval (CI), respectively. The treatment effect for the respective outcome measures was considered potentially worthwhile if the MDC was contained within the 95% CI of the mean between-group difference [5]. Activity level, GROC, and PASS outcomes were reported descriptively. Decision criteria for progression to a full-scale RCT was based on: i) all feasibility criteria being met, or reasonable recommendations to achieve criteria in future trials and ii) presence of a potentially worthwhile treatment effect for knee-related QoL, symptoms, and function.

## Results

### Feasibility

**Fig. 1:**
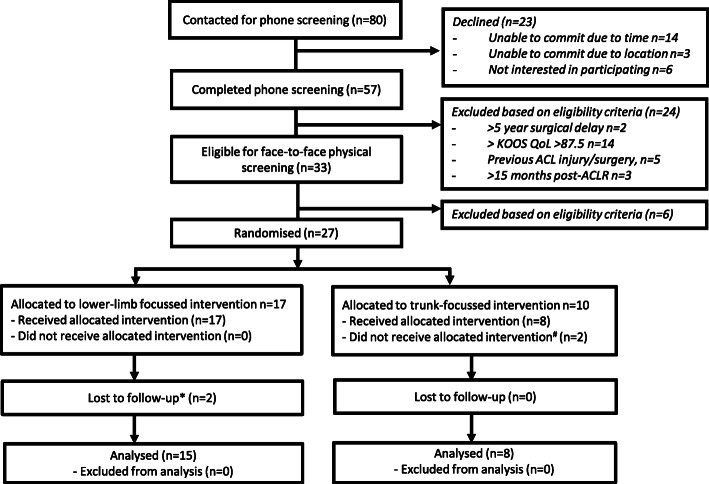
Flow of participants through the study. KOOS-QoL=Knee injury and Osteoarthritis Outcome Score Quality of Life subscale; ACL=anterior cruciate ligament; ACLR=anterior cruciate ligament reconstruction. ^#^ *n*=2 unable to find appointments to suit work/study commitments. *n=1 severe increase in knee pain, *n*=1 unable to commit to requirements

All feasibility criteria were met, or reasonable recommendations could be made to achieve the criteria in future trials (Table 2). Eighty people expressed interest in participation via response to letters from their surgeon (*n*=55), or advertisements on social media and La Trobe University (*n*=25) over a 9-month period. 72% (*n*=57) agreed to be screened, with 47% of those screened (*n*=27) deemed eligible (Fig. 1). The results of each aspect of feasibility are summarised in Table 2, with the detailed feedback provided by participants at follow-up provided in Additional file 4.

**Table 2:** Feasibility outcomes

	CRITERION	LOWER-LIMB GROUP (***n***=17)	TRUNK GROUP (***n***=10)	Proceed	Proceed with amendments
**Recruitment, retention, adherence**					
*Recruitment rate*	> 4 per month	3 participants per month		No	Strategies to increase recruitment rate
*Enrolment rate*	> 90%	100% completed baseline assessment, were enrolled and randomised		Yes	
*Drop-out rate*	< 20%	n=2 (12%)^a^	n=2 (20%)^b^	Yes	
*Physiotherapy attendance*	> 80%	Mean = 89% of intended 8 sessions	Mean = 86% of intended 8 sessions	Yes	
*Exercise adherence*	> 80%	52% of sessions completed	48% of sessions completed	No	Strategies to increase adherence
**Study protocol acceptability**					
*Eligibility rate*	1 in 3	47% of interested participants were eligible		Yes	
*Acceptability of intervention to physiotherapists*	Descriptive	• Training and supportive material sufficient • Reflected clinical practice, but time allocation insufficient		No	Appointments > 30 min or provide additional more frequent appointments
*Acceptability of intervention to participants*	Descriptive	• Appointment duration/frequency, and facilities appropriate. • Interventions were credible and acceptable		Yes	
*Barriers to adherence*	Descriptive	Work, study and family commitments, lack of motivation, boredom with exercises		No	Strategies to address common barriers
**Adverse Events**					
*Injury or illness*	< 20%	*n*=4 (24%) unrelated to intervention^c^	Nil	Yes^d^	
*Pain during/after exercise*	< 2/10	Mean pain < 2/10 for each participant (across all sessions)		Yes	
**Randomisation integrity**					
*Integrity of blinding*	90%	Assessor unblinded for n=1	1 participant (medical professional) knew they were in the “control” group	Yes	
*Group contamination*	0%	Nil	Physiotherapists reported often discussing patient-specific topics	No	Control education difficult to standardise for control groups
**Acceptability of outcomes**					
*Time to collect data*	< 90 min	Baseline and follow-up assessments were completed in 60–90 min		Yes	
*Completeness of PROs*	> 90%	All 23 participants who finished the trial completed PROs, with no missing data		Yes	
*Completeness of functional performance outcomes*	> 90%	16% (n=4) did not complete follow-up^e^	10% (*n*=1) did not complete follow-up (located internationally)	No	Consider PROs as primary outcome for complete data
*Adherence monitoring*	Descriptive	• 23 used PhysiApp**©** and 2 used paper diaries • Enjoyed the accountability and motivation PhysiApp**©** provided, and the videos for exercise technique • Inconsistently used PhysiApp**©** to record adherence (i.e. technical issues, or forgot to use the app as they knew their program)		No	Strategies to increase adherence to data entry (e.g. incentives, interactive features such as benchmarking, education)

^a^ *n*=1 severe increase in knee pain, *n*=1 unable to commit to requirements

^b^ *n*=2 decided they could not commit to the trial before commencing interventions

^c^ *n*=1 severe increase in knee pain (group fitness class); *n*=2 hamstring strains (sprint training, sprint in basketball match); n=1 ankle sprain (football training)

^d^ Consider high-speed running and sport-specific programs to reduce risk of other soft tissue injuries

^e^ *n*=2 could not complete hop-test (recovering from ankle sprain and hamstring strain), n=2 could not attend (located internationally, work commitments).

### Participant characteristics

The trunk-focussed group had a higher proportion of men, a higher proportion participating in Level 1 or 2 sports pre-injury, and higher proportion of concomitant meniscal surgery at the time of ACLR (Table 3).

**Table 3:** Participant characteristics at baseline

	Lower-limb focussed group (***n***=17)	Trunk-focussed group (***n***=10)
**Age**, mean±SD years	34±12	33±12
**Sex**, no. (% male)	6 (35%)	7 (70%)
**Body mass index**, mean±SD kg/m^2^	24.7±2.8	25.4±3.8
**Pre-injury activity level**^a^**,** no. (%)		
Level 1 or 2	19 (53%)	10 (100%)
Level 3	6 (35%)	0 (0%)
Level 4	2 (12%)	0 (0%)
**Concomitant meniscal procedure**^b^**,** no. (%)	9 (53%)	3 (30%)
**Ceased supervised PT,** median (IQR) months	7 (6)	6 (5)

*SD* Standard deviation, *PT* Physiotherapy

^a^ According to Grindem classification system [32]. Level 1=pivoting/jumping/hard cutting sports (e.g. football), Level 2=pivoting/jumping sports but less intense cutting (e.g. volleyball), Level 3 sports=straight line activities (e.g. running, weight-lifting), and Level 4=sedentary

^b^ at the time of ACLR, reported by participants at baseline assessment

### Patient-reported outcomes

The desired treatment effect for KOOS-QoL (improvement > 8–10 points) and ACL-QoL (improvement > 12 points) was contained within the 95% CI (Table 4). Additional file 5 reports the individual treatment responses for KOOS-QoL and ACL-QoL, and proportion with improvements greater than the MDC. The KOOS-Pain, KOOS-Sport, KOOS_4_, GROC and PASS indicated both groups improved, potentially to greater extent in the lower-limb focussed group (Table 4). The MDC was contained within the 95% CI for KOOS-Pain, KOOS-Sport and KOOS_4_ (Table 4). Majority (87%) were at least “better” for knee function and knee pain in the lower-limb focussed group, compared to 50 and 75% respectively in the trunk-focussed group. Satisfaction (PASS question) with current knee function improved in the lower-limb focussed group from 27 to 67%, but remained the same in the trunk-focussed group (63%).

**Table 4:** Secondary outcomes at baseline and follow-up~

	Lower-limb focussed intervention (***n***=15)			Trunk-focussed intervention (***n***=8)			Lower-limb vs trunk	Previously published MDC values
	Baseline	Follow-up^	Change	Baseline	Follow-up^	Change	mean difference in change* (95%CI)	
**ACL-QoL**	45±20	64±20	20±17	56±9	78±16	22±13	−2.5 (−18.2 to 13.2)	12 points (Lafave et al., 2017)
**KOOS-QoL**	39±20	62±23	23±25	52±14	67±19	16±12	7.1 (−12.3 to 26.4)	8 to 10 points (Roos & Lohmander, 2003)
**KOOS-Symptoms**	68±23	74±19	7±17	81±9	90±9	9±7	−2.0 (− 15.7 to 11.6)	8 to 10 points (Roos & Lohmander, 2003)
**KOOS-Pain**	77±15	86±12	9±14	90±7	92±8	2±7	6.7 (−4.0 to 17.9)	8 to 10 points (Roos & Lohmander, 2003)
**KOOS-Sport**	57±24	77±22	20±25	76±1	83±22	8±13	12.1 (−7.9 to 32.0)	8 to 10 points (Roos & Lohmander, 2003)
**KOOS**_**4**_	60±17	75±17	15±18	75±9	83±14	9±7	5.9 (−7.9 to 19.8)	8 to 10 points (Roos & Lohmander, 2003)
**ACL-RSI**	36±18	53±22	17±18	41±18	67±24	26±22	−9.2 (−27.2 to 8.7)	19 points (Kvist et al., 2013)
**Single-hop**								
ACLR (cm)	65±42	97±33	33±34	108±39	115±42	8±9	24.1 (−5.9 to 54.1)	14 cm (Kockum & Heijne, 2015; Reid et al., 2007)
Contralateral (cm)	93±30	106±32	14±18	116±25	120±34	4±15	9.6 (−8.9 to 28.2)	14 cm (Kockum & Heijne, 2015; Reid et al., 2007)
LSI (%)	59±38	88±11	29±37	90±21	94±12	4±10	15.5 (−27.7 to 58.7)	8% (Kockum & Heijne, 2015; Reid et al., 2007)
**Side-hop**								
ACLR (reps)	8±8	16±12	9±9	20±13	29±19	9±8	−0.6 (−9.4 to 8.7)	11 reps (Kockum & Heijne, 2015; Reid et al., 2007)
Contralateral (reps)	9±9	17±12	8±10	23±16	31±21	9±15	−0.5 (−13.4 to 12.2)	11 reps (Kockum & Heijne, 2015; Reid et al., 2007)
LSI (%)^a^	71±42	82±29	11±28	73±13	94±14	21±18	−10.4 (− 43.1 to 22.2)	^~^ 10% (Kockum & Heijne, 2015; Reid et al., 2007)
**One-leg rise**								
ACLR (reps)	17±16	33±15	17±14	27±19	34±20	7±11	9.9 (−4.1 to 23.9)	Not available
Contralateral (reps)	25±19	35±15	10±14	31±15	34±15	4±7	6.4 (−6.5 to 19.3)	Not available
LSI (%)^b^	67±57	98±9	31±54	68±32	83±47	16±32	15.7 (−40 to 71.5)	Not available

*ACLR* Anterior cruciate ligament reconstructed limb, *ACL-RSI* ACL Return to Sport Index, *ACL-QoL* Anterior Cruciate Ligament Quality of Life questionnaire, *CI* Confidence interval, *KOOS* Knee injury and Osteoarthritis Outcome Score, *LSI* Limb symmetry index, *MDC* Minimal detectable change, *SD* Standard deviation, *QoL* Quality of life

* Positive value indicates between-group differences are in favour of the lower-limb focussed group

^*n*=3 participants did not complete functional performance follow-up (could not attend due to being overseas, or work commitments). An additional 2 participants did not complete the single-hop and side-hop tests as they were recovering from adverse events (*n*=1 hamstring strain and *n*=1 ankle sprain).

^a^ *n*=3 not included for LSI calculation at baseline (3 in lower-limb focussed), and n=3 not included at follow-up (2 in lower-limb focussed, 1 in trunk-focussed), as unable to perform a valid score on either limb

^b^ *n*=5 not included for LSI calculation at baseline (n=3 in lower-limb focussed group and *n*=2 in trunk-focussed group), and *n*=1 (in trunk-focussed group) not included at follow-up, as unable to perform a valid score on either limb

~Values are mean+/-SD unless otherwise indicated

### Functional performance outcomes

The MDC (where available) was contained within the 95% CI for all functional performance tests, except for the side-hop ACLR limb performance (Table 4). Additional file 5 demonstrates the proportion of participants who have improvements greater than the MDC, and highlights the large individual variation in change in functional performance in both groups.

## Discussion

The results of this study suggest it is worthwhile proceeding to a large-scale RCT evaluating the effectiveness of a physiotherapist-guided lower-limb focussed exercise-therapy and education intervention for young adults who have persistent symptoms 1-year post-ACLR. All feasibility criteria were either met, or reasonable recommendations could be made to achieve the criteria in future trials. Additionally, worthwhile treatment effects were observed in participants receiving the lower-limb focused intervention for knee-related symptoms, function and QoL.

### Feasibility: recruitment, retention, attendance and protocol acceptability

Of those screened, almost half (47%) were eligible, and we achieved a modest recruitment rate (3 per month). For a large-scale RCT, the number of participating surgeons (and study advertising) would need to be increased, which is possible due to the large number of ACLRs performed each year [50]. Although all eligible participants were willing to enrol, two participants did not commence the intervention, and two others dropped out during the intervention, resulting in an overall drop-out rate of 16%, which is considered acceptable [28]. Physiotherapy attendance was high (86 to 89%), which is similar to previous physiotherapist-guided exercise-therapy RCTs (> 80%) for lower-limb musculoskeletal conditions in young adults [2, 41]. Suggestions during feedback from drop-outs and those who attended less than 80% of study appointments (*n*=5) aligns with previous reported strategies to maintain attendance – i.e. increasing appointment availability after hours, exercise variety, and strategies to increase motivation [58]. These strategies, in addition to consideration of telehealth appointments, and multiple clinic locations might reduce drop-outs and improve attendance in future trials. Longer appointment duration or more frequent appointments may be required in future trials to provide physiotherapists with sufficient time to review exercise programs and provide education. Those with persistent symptoms may also require additional informational and emotional support, considering their knee-related QoL, symptoms, and function is considerably lower than most patients 1-year post-ACLR [55, 65].

### Feasibility: adherence to the unsupervised exercise-therapy program

According to Physitrack© adherence data, only half of the prescribed unsupervised exercise-therapy program sessions were completed. However, these data are likely to under-estimate true exercise adherence in this trial, as participants reported inconsistently entering their adherence data in Physiapp© due to technical difficulties, and rarely using the app once familiar with the exercises. Regardless of true adherence rates, participants did report typical barriers to exercise adherence, including other commitments (work, study and family), and reduced motivation [30, 73]. Exercise adherence rates were lower than previous reports for rehabilitation during the first 6-months following ACLR (75 to 80%) [8, 59]. This may reflect the burden of exercise-therapy on participants who have already endured unsuccessful rehabilitation with the physical, mental and time commitment it entails. Strategies to increase adherence (to the unsupervised exercise-therapy program and monitoring system) may include goal setting [77], incentivisation, supervised group classes, or alternate exercise options (e.g. non-gym based) [30]. Personalised adherence monitoring data collection methods, including paper diaries, email or text questionnaires, and strategies to maintain engagment with apps (e.g. positive reinforcement, benchmarking) should be considered.

### Feasibility: adverse events, integrity of group allocation, acceptability of outcomes

Two participants sustained hamstring strains in their ACLR limb, and one sustained an ankle sprain as they returned to sporting activities. Graded return to high-speed running protocols should be emphasised in future trials to reduce soft tissue injury risk, especially given ACL injury is a well-recognised risk factor for hamstring strain [31]. Future trials should include strategies to maintain participant blinding (e.g. control interventions that are credible), and sufficient study personnel to maintain at blinding of assessors. Completion of follow-up PROs (100%) and functional performance measures (81%), suggests PROs may be the most appropriate outcome tool to optimise completion in future trials.

### Future recommendations: treatment effects for knee-related QoL

A worthwhile effect (>MDC) was observed for both the lower-limb and trunk-focussed interventions for the KOOS-QoL and ACL-QoL. Our results are consistent with other trials which comparing two types of physiotherapist-guided exercise interventions [2, 43]. The trunk-focussed group was hypothesised to have minimal effect on knee-related QoL, but greater trunk strength and endurance may improve perceived performance in sport and work-related activities, resulting in better QoL. Indeed, the trunk-focussed group had improvements in self-reported function (KOOS-Sport) which equalled the MDC (8 points). Improvements in knee-related QoL may be more strongly influenced by education (provided in both groups). In both groups, physiotherapists were able to educate participants and address psychological factors (e.g. kinesiophobia, fear, confidence), which are known determinants of adherence, recovery and self-reported outcomes after sports-related knee injury [30, 70]. The physiotherapists reported discussing patient and ACL-specific topics with the trunk-focussed participants, although directed not to do so in the study protocol, which may have had a direct or indirect effect on knee-related QoL. Future RCTs may consider evaluating the effects exercise-therapy and education with a comparator that better reflects usual care (e.g. self-directed exercise), and/or including a wait-list control. Given health-professional delivered education alone may be effective in young people with persistent knee pain [22], future trial designs might compare: (i) exercise-therapy versus education alone; and/or (ii) exercise-therapy with and without education, to guide interventions for those at risk of post-traumatic OA after ACLR.

### Future trial recommendations: treatment effects for knee-related function

While the study was not powered to detect between-group differences, the lower-limb focussed group had greater improvements in KOOS-Sport, single-hop and one-leg rise likely to be clinically meaningful in a larger trial. However, these findings should be interpreted with caution due to the wide CIs, and between-groups differences at baseline particularly for the ACLR limb functional performance. In the lower-limb focussed group, the LSI improvements for the single-hop (29%) were larger than those in the ACL-SPORTS trial (10%) with a similar lower-limb focussed intervention [2]. This larger improvement we observed may be due the lower baseline function of our participants compared to the ACL-SPORTS trial where all participants had already achieved ≥80% LSI, begun running, and had no pain [2]. Future studies should also consider that LSI improvement can reflect worsening contralateral limb function, rather than improved ACLR limb function [54]. Therefore, it is important to note that in the current interventional study, LSI improvements occurred alongside clinically meaningful improvements in both limbs, indicating the increase in LSI was due to *greater* improvement in the ACLR limb. Given poor function on hop-tests at 1-year post-ACLR may be associated with an increased risk of future OA [53, 57] and re-injury [33], addressing persisent functional deficits may be an important step forward in secondary prevention of post-traumatic knee OA. Considering the influence of the lower-limb focussed intervention in this study on OA risk factors, future larger-scale trials should consider longer-term follow-up and include imaging assessment to determine structural joint trajectory and relationship with symptoms [19], physical activity monitoring, healthcare utilisation, and cost-effectiveness evaluation.

### Recommendations for future trials: intervention content and format

Despite improvements, knee-related function and QoL remained lower than uninjured normative values [4, 42, 52], and satisfaction with knee function was less than 70% in the lower-limb focussed group at follow-up, indicating that the lower-limb focussed intervention could be improved for future trials. Future trials may consider a longer intervention, with more frequent supervised sessions (either 1-to-1 or group exercise classes) to provide further opportunity for education and exercise progression to address persistent impairments. Some lower-functioning patients with persistent symptoms at 1-year post-ACLR may not wish to return-to-sport and have reduced motivation to complete plyometric and agility exercises. Future trials should consider a pragmatic individualised approach to exercise prescription, allowing the physiotherapist to choose from a set of exercises according to the patient’s needs and goals. Isolated quadriceps exercises (e.g. knee extension) may be important to include in future interventions for those with persistent symptoms, due to the known associations between quadriceps weakness and development of symptomatic OA in the general population [20].

### Limitations and recommendations for full-scale RCT

Given this was a pilot feasibility study, it did not have adequate power to establish superiority of one intervention over the other. Further, the lower-limb focussed group started with worse knee-related QoL and function, compared to the trunk-focussed group (Table 4), allowing greater room for improvement compared to the trunk-focussed group. A larger sample size would likely reduce baseline between-group heterogeneity. Future large-scale RCTs, including stratification for factors that may affect baseline status or treatment response (e.g. sex) [21] are now needed. Many participants (> 50%) had a surgical review during the trial, and were given “clearance for return-to-sport”, which may have improved PROs in both groups. Future trials should regularly (weekly or monthly) monitor all types of physical activity completed during the intervention period. Despite these limitations, this was a pilot feasibility study, with the purpose of recognizing improvements that could be made to the study design and protocols for future trials. Consistent with other ACLR cohorts [62] and RCTs [2], there was large individual variation in both groups for baseline scores (i.e., SDs) and changes between baseline and follow-up for all primary outcomes (Additional file 5). We did not assess lower-limb or trunk strength so we cannot indicate if the improvements in functional performance or PROs were mediated by strength increases. Future trials should include muscle capacity (strength, power) testing, to also ensure that adequate loading and progression has occurred to stimulate muscle capacity improvements [14]. Participating surgeons and clinic locations were limited to metropoliation Melbourne and Hobart. Future trials should be aware recruitment, eligibility, attendance and adherence rates may differ in other settings.

## Conclusion

A large-scale trial to evaluate the effectiveness of a physiotherapist-guided exercise-therapy and education program for individuals with persistent symptoms at 1-year post-ACLR is feasible. All feasibility criteria were met, or reasonable recommendations could be made to achieve the criteria in future trials. Strategies to increase recruitment rate, adherence to exercise and data completion are required. Potential worthwhile treatment effects for knee-related QoL, symptoms and function were observed, indicating a fully-powered RCT may detect a clinically meaningful effect.

## Supplementary Information

**Additional file 1.** CONSORT Checklist.

**Additional file 2.** Physiotherapist manual: exercise-therapy and education protocols.

**Additional file 3.** Lower-limb focussed and trunk-focussed exercise-therapy and education interventions website https://task.trekeducation.org/.

**Additional file 4.** Feedback from study participants on exercise program content, structure and delivery methods.

**Additional file 5.** Secondary outcomes additional detail.

## Data Availability

The datasets used and/or analysed during the current study are available from the corresponding author on reasonable request.

## Abbreviations

ACL: Anterior cruciate ligament
ACLR: Anterior cruciate ligament reconstruction
ACL-QoL: ACL quality of life survey
ACL-RSI: ACL Return to Sport Index
BMI: Body mass index
CI: Confidence interval
CERT: Consensus on Exercise Reporting Template
CONSORT: CONsolidated Standards of Reporting Trials
GROC: Global rating of change
KOOS: Knee injury and Osteoarthritis Outcome Score
LSI: Limb symmetry index
MDC: Minimal detectable change
OA: Osteoarthritis
PASS: Patient acceptable symptom state
PROs: Patient reported outcomes
RCT: Randomised controlled trial
SD: Standard deviation
TIDieR: Template for Intervention Description and Replication guidelines
QoL: Quality of life
