# Supplementary material for: Exercise-therapy and education for individuals one year after anterior cruciate ligament reconstruction: a pilot randomised controlled trial
Source: BMC Musculoskelet Disord. 2021 Jan 11;22:64. doi: 10.1186/s12891-020-03919-6 (PMC7802328; doi:10.1186/s12891-020-03919-6)
Supplement: Supplementary file 2 — Additional file 2. Physiotherapist manual: exercise-therapy and education protocols. [file 12891_2020_3919_MOESM2_ESM.pdf]

PROJECT DETAILS

An exploration of the feasibility of a randomised clinical trial for physiotherapy intervention in patients at high risk of early-onset knee osteoarthritis and symptomatic decline following anterior cruciate ligament reconstruction.

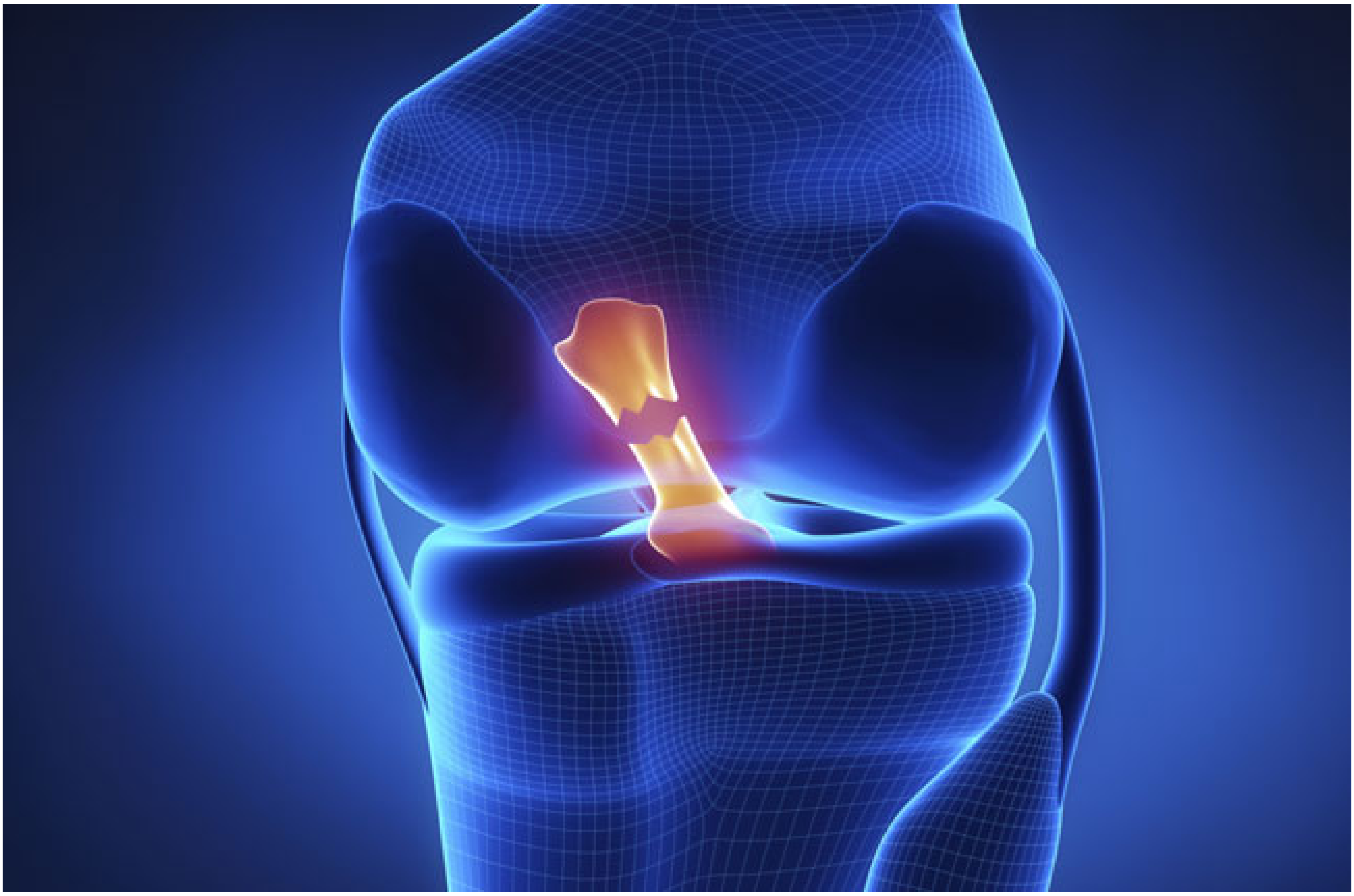

Investigators: Ms Brooke Patterson, Professor Kay Crossley, Dr Adam Culvenor, and Dr Christian Barton.

If you have any questions relating to the project please contact Chief Investigator – Professor Kay Crossley.

This project is approved by the La Trobe Human Ethics Committee (HEC-16-077)

Please remember – investigator Brooke Patterson is blinded to patient group

LA TROBE UNIVERSITY | Sport and Exercise Medicine Research Centre

INTRODUCTION

This exercise-therapy and education protocol is a guide for treating physiotherapists in a pilot randomised controlled trial who are treating individuals who have undergone ACL reconstruction 12-15 months prior and have been identified at risk of early osteoarthritis progression and symptomatic decline. The guide contains both our intervention (lower-limb focussed) and control (trunk-focussed) protocols.

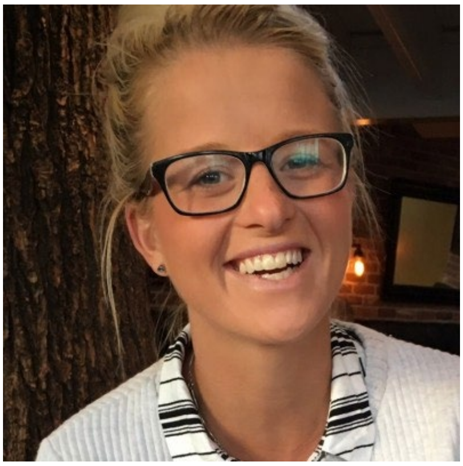

The lower-limb focussed program *(note patient-facing name: "Kangaroo")* is based on the best available evidence and by a group of experienced clinician researchers in the ACL and musculoskeletal field. People following ACLR continue to have persistent lower -imb neuromuscular deficits, do not return to sport, and have reduced quality of life. Rehabilitation requires great commitment from the patient and the therapist, and there are multiple areas to work on and monitor.

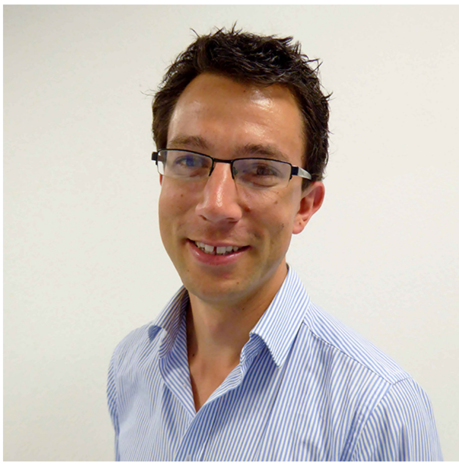

Every patient is different and the purpose of this guide is not to produce a recipe, as it allows for varying levels of ability and goals. We have chosen key areas or "tasks" that we believe are most important to recovery in this population, with each task having multiple phases to allow for individualisation.

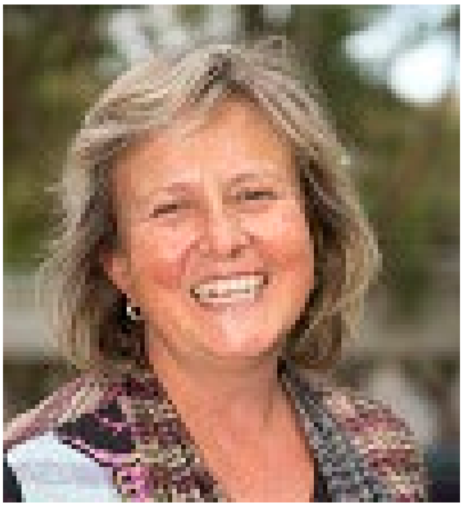

This exercise therapy and education protocol for the trunk-focussed group *(note patient facing name: "Devil")* is outlined on Page 17-19

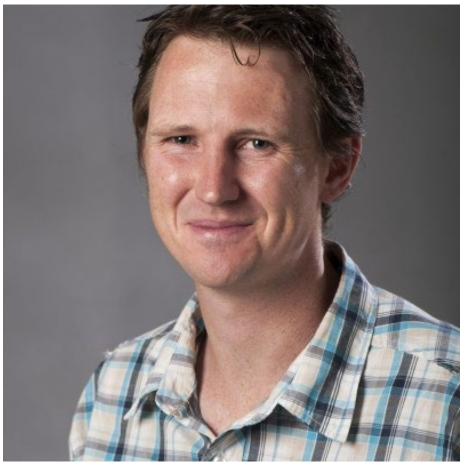

Brooke Patterson   Dr Adam Culvenor

Professor Kay Crossley   Dr Christian Barton

La Trobe University Sports and Exercise Medicine Centre

For further information and many more sports medicine related topics visit: http://semrc.blogs.latrobe.edu.au

PHYSIOTHERAPY PROGRAM: TASK PROJECT (Group: Kangaroo) Participant ID:

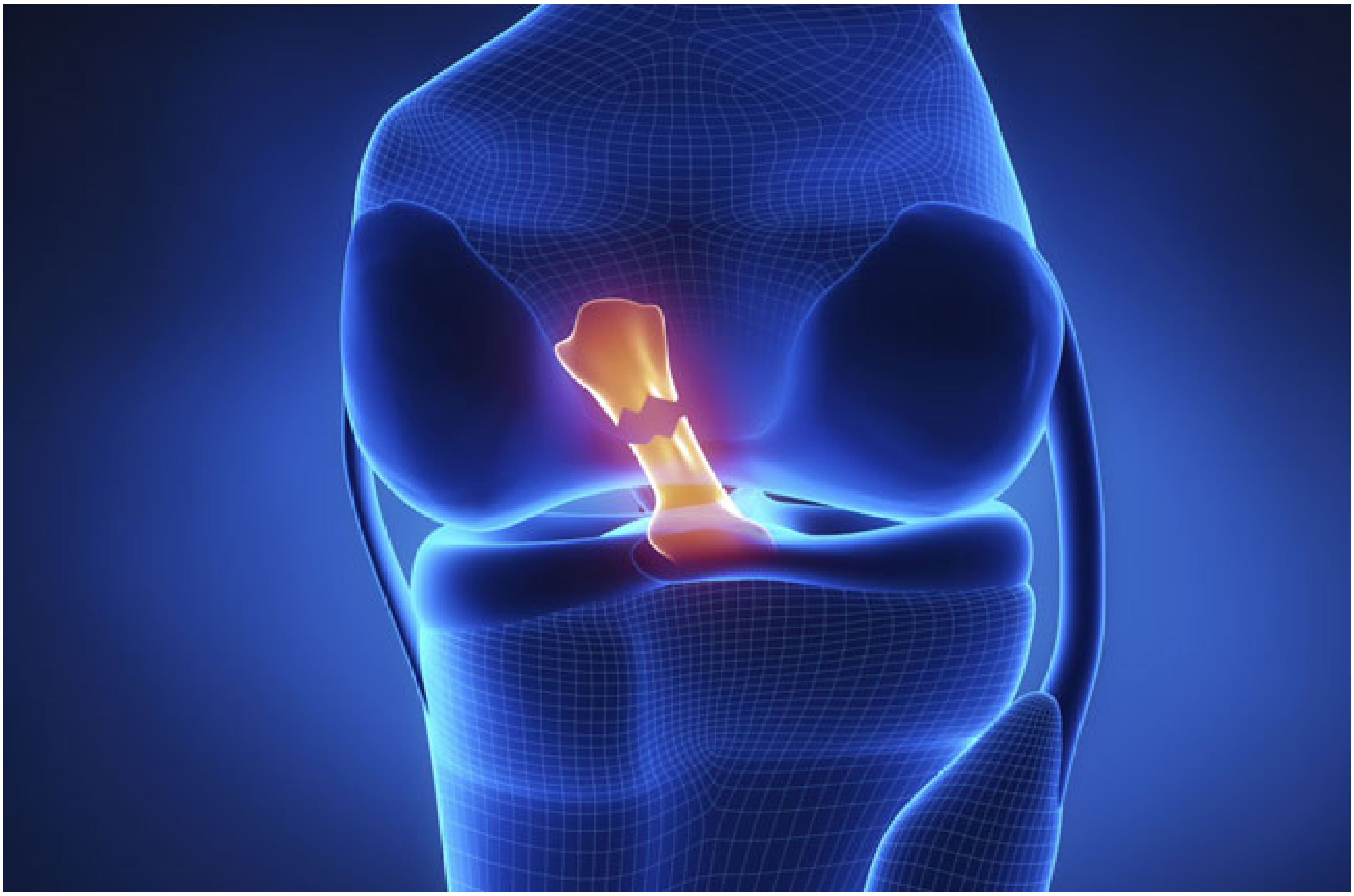

This booklet contains:

- Exercise protocol and progressions
- Physiotherapist clinical notes and attendance record
- Educational resources and handouts
- **Other resources you may need:**
- Administrative procedures physiotherapy handbook

OVERVIEW OF PROGRAM
(lower-limb focussed ntervention)

1. General

This exercise therapy and education program is designed for every patient to progress at his or her own pace and ability. Patients are to attend 8 face-to-face physiotherapy sessions of 30 minutes duration. Therapists are to use the treatment sessions to progress through each "task" (target area) individually ensuring that the patient is challenged and working towards a strength and/or power dosage as recommended by resistance training principles.

2. Exercise therapy protocol

These “tasks” comprise of various muscle groups and movements that must be targeted after ACL reconstruction. Each task has at least 3 phases of difficulty, and guides for progression criteria. Patients can be on different phases of difficulty for different tasks. I.e. Phase 1 of balance, and Phase 3 of calf.

TASK 1: Movement rretraining (walk – run – jump- land – cut)

TASK 2: Squat

TASK 3: Balance

TASK 4: Hip stabilisers

TASK 5: Calf

TASK 6: Trunk

TASK 7: Hip extensors

TASK 8: Cardiovascular/sport

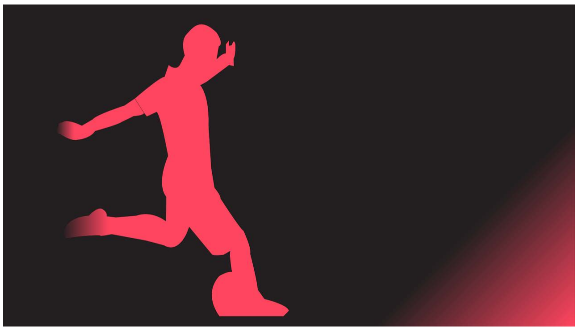

It may not be appropriate for patients to be completing exercises in all tasks - use clinical reasoning to prescribe exercises based on the patient’s needs, goals and level of adherence.

3. Independent Exercise Program

Encourage patients to complete the exercise-therapy program a minimum 3x week. Use clinical reasoning if appropriate to prescribe more or less and combine with sporting activities.

4. Manual therapy

If appropriate you may perform manual therapy if impairments such as pain, swelling, range of motion or joint mobility is affecting the patient's ability to perform the exercises. Please follow the treatment algorithm on Page 14.

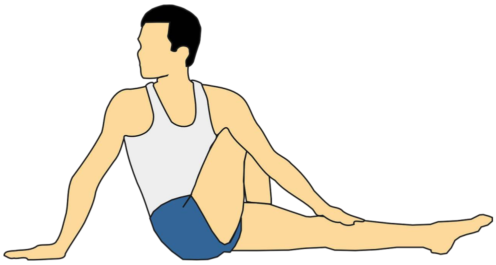

Mobility and stretching exercises may be appropriate if you deem range of motion or muscle length is affecting the patient's ability to perform an exercise (i.e. hip flexor/quadriceps length affecting bridge

5 Education

It is important to educate the patients on the level of commitment required to benefit from a resistance training program, achieve adequate physical and psychological criteria to return to sport, and independently manage their knee joint health in the long term. Remind patients of the following expectations, and provide them with the handouts (Page20 to 34) as you deem appropriate.

Time:
Exercises at least 3 x week.
Dependent on the level of recovery, ability, and goals of the individual.

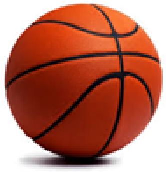

Equipment:
When they will need to access the gym.

Return to sport:
Does the patient want to get back to previous level of activity? When?
Based on achievement of specific goals (strength, level of functioning), not time.

Clinicians should follow the guide for return to running and return to sport outlined on Page 35-36. Each of the outcome measures related to the criteria are outlined on Page 37-44.

Provide patients with the leaflets on Page to 20-34 and provide face-to-face education as appropriate.

TASK 1: MOVEMENT RETRAINING					Physiotherapy Log
	Straight Line	Landing	Lateral	Multidirectional	
**Phase 1**	“Walking drills”<br>Normal gait	“Landing technique”<br>Walk through and teach key points	“Lateral movement technique”<br>Walk through and teach key points	See Video: Cutting technique<br>Walk through and teach key points	Session (_): Date:<br>Phase + Dosage:
**Dosage***	Complete 10 minutes of 1:1 movement technique with physio<br>At home: 10 sets of 5 (rest 30s), daily				
**Phase 2**	“Running program”<br>(see Task 8)	“Jump series”<br>(Increase height/length) with:<br>- On spot<br>- Forwards<br>- On to step<br>- Off step (progress to with a jump)	“Lateral movement technique”<br>Progress through the following<br>- Step lateral, shift weight and touch cone<br>- Moving sidesteps – without resistance, with theraband, increase speed<br>- Bounding side to side	“Agility Program”<br><br>45 – 90 – 135 – 180 degree cuts	Session (_): Date:<br>Phase + Dosage:<br><br>Session (_): Date:<br>Phase + Dosage:
**Dosage***	As per Task 8 graduated	5 sets of 5 reps max effort BW (rest 60s) max effort. 3 x week		As per program on Task 8 graduated	
**Phase 3**	Sprinting program (see Task 8)<br><br>“Running program”<br>PHYSITRACK	“Hop series”<br>- as per jump series above	“Side jump series”<br>- Increase speed/decrease contact time<br>- Over hurdles/step	Multidirectional and unanticipated<br>– Including individual & partner skill drills	Session (_): Date:<br>Phase + Dosage:<br><br>Session (_): Date:<br>Phase + Dosage:
**Dosage***	As per Task 8 graduated	5 sets of 5 reps max effort BW (rest 60s. 3 x week)		As per program on Task 8 graduated	
**Phase 4**	Acceleration/<br>Deceleration/<br>program (see Task 8)<br><br>“Running program”<br>PHYSITRACK	Any advanced landing specific to sport and integrate sport specific skills	“Side hop series”<br>- Increase speed / decrease contact time<br>- Over hurdles/step	Integrate into non contact training drills	Session (_): Date:<br>Phase + Dosage:<br><br>Session (_): Date:<br>Phase + Dosage:
**Dosage***	As per Task 8 graduated	5 sets of 5 reps max effort BW (rest 60s) 3 x week		As per program on Task 8 graduated	Session (_): Date:<br>Phase + Dosage:

*Pass once completed set dosage + correct technique/functional alignment/area of fatigue, nil response of pain (<2/10) or swelling.

**TASK 2: SQUATS**		Physiotherapy Log
**Phase 1**	Wall Sit	Session (_): Date:<br>Phase + Dosage:
	Progress:<br>- Depth 30 - 90 degrees**	
**Dosage**	3 sets of 60s isometric (rest 60s) BW. 3 x week	Session (_): Date:<br>Phase + Dosage:
Progress to Phase 2*	Able to DL BW Squat x 15 <2/10 pain<br>OR Wall Sit to 90 degrees	
**Phase 2**	Squatting Series**	
	Double leg Options<br>- "Squat"<br>- "Squat" – dumbells<br>"Squat" – barbell back squat	Session (_): Date:<br>Phase + Dosage:
	Single Leg Options<br>- "Single Leg Rise" assisted<br>- "Single Leg Rise" unassisted<br>- "Single Leg Rise" with external load (5 - 15kg)<br>- "Single leg squat" +/- load	Session (_): Date:<br>Phase + Dosage:<br>Session (_): Date:<br>Phase + Dosage:
**Dosage 3 x week**	3 x 10-12 reps (3s down, 0 hold, 2s up) (60s rest)<br>Fatigue at 12 reps<br>Can be doing DL and SL exercise coinciding<br>3 x week : Double leg<br>3 x week: Single leg	Session (_): Date:<br>Phase + Dosage:
Progress to Phase 3*	Able to squat DL Body Weight external load	
**Phase 3**	Squatting Series (Power)**	
	As above different dosage<br>Advanced – jump / hop with smith machine bar / load	Session (_): Date:<br>Phase + Dosage:
**Dosage 3 x week**	5-10 repetitions, 3-5 sets (2s down, 0 hold, 1s up) (60-90s rest) Progress load as appropriate<br>Note: Patient can be doing DL and SL strengthening at the same time. For example, Phase 2 (double or single) exercise in the same session or 2 x week Phase 2, and 1 x week Phase 3	Session (_): Date:<br>Phase + Dosage:

*Pass once completed set dosage + correct technique/functional alignment/area of fatigue, nil response of pain (<2/10) or swelling.
**Depth as appropriate for patient (their anatomy/ROM, goals, pathology)

TASK 3: BALANCE		Description of Exercise	Physiotherapy Log
**Phase 1**	"SLS" (Eyes Open -> Eyes Closed)	Focus on early awareness of:<br>- Foot control<br>- Gluteal activation and pelvic position<br>- Alignment of shoulder, hip, knee, foot	Session (_): Date:<br>Phase + Dosage:
**Dosage**	3 x 30s hold each side (0s rest). Daily#		
Progress to Phase 2*	15s eyes closed each side good control, no loss of balance		Session (_): Date:<br>Phase + Dosage:
**Phase 2**	"SLS Bosu" (Eyes Open -> Closed)<br>Bosu ball/foam/Pillow at home		
**Dosage**	3 x 30s hold each side (0s rest). Daily#		
Progress to Phase 3*	15s eyes closed each leg good control, no loss of balance		Session (_): Date:<br>Phase + Dosage:
**Phase 3**	"Balance and reach"<br>- Hand to cones /throwing task<br>- Toe to cones<br>- Progress distance appropriate to patient size<br>- Progress to foam		Session (_): Date:<br>Phase + Dosage:
**Dosage**	3 x 10 touches each side (2s down 2s up) (0s rest). Minimum 3 x week		
Progress to Phase 4*	10 each side with good control, no loss of balance		Session (_): Date:<br>Phase + Dosage:
**Phase 4**	"Advanced Balance series 1 or 2" (can vary it)<br>i) Various balancing on ½ foam roller / beam<br>ii) +/- Skills<br>iii) Up to discretion of physio – make sport specific<br>Alternatives on Physitrack – "curtsy slide' "Jump on off bosu"	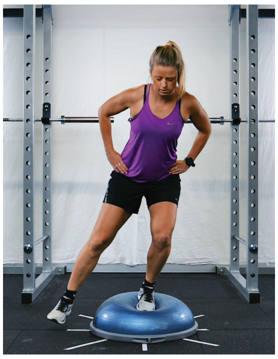	Session (_): Date:<br>Phase + Dosage:
**Dosage**	3 x 60s duration (60s rest). Minimum 3 x week		
Ongoing progression *	Continue to challenge patient as appropriate or specific to their sport.		Session (_): Date:<br>Phase + Dosage:<br>Session (_): Date:<br>Phase + Dosage:

*Pass once completed set dosage + correct technique/functional alignment/area of fatigue, nil response of pain (<2/10) or swelling.

#In Physitrack "add exercise" go to appropriate balance exercise and click add to specific "off" days – usually 2,4,6,7.

**TASK 4: Hip**		Description of Exercise	Physiotherapy Log
**Phase 1**	Side-lying hip abduction	*Emphasise posterior pelvic tilt*<br><br>*"Heel behind hip"/don't let leg drift forward*<br><br>*trunk position/don't let back push into ground*	Session (_): Date:<br>Phase + Dosage:
	Progress:<br>- Theraband resistance (Yellow – Green – Blue) and length		
**Dosage**	3 x 10-12 (3s up, 1s hold, 3s down) each side to 45 degrees (swap 0s rest)<br>Load: BW or theraband to fatigue at 12 reps/12RM<br>If BW - 5 x week<br>Theraband – 3 x week		Session (_): Date:<br>Phase + Dosage:
Progress to Phase 2*	3 x 10-12 blue theraband (15cm length when tied) to 30 degrees		Session (_): Date:<br>Phase + Dosage:
**Phase 2**	Standing Hip Abduction Resisted	*Light upper limb support allowed*	
	Progress:<br>- Theraband resistance (Yellow – Green – Blue) and length	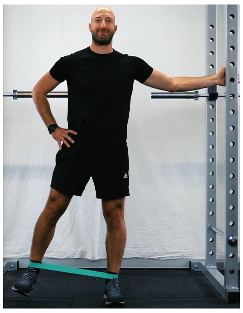	
Dosage	3 x 10-12 (3s out, 1s hold, 3s in) each side to 30 degrees (swap 0s rest)<br>Load: BW or theraband to fatigue at 12 reps/12RM<br>3 x week		Session (_): Date:<br>Phase + Dosage:
Progress to Phase 3*	3 x 10-2 blue theraband (15cm length when tied) to 30 degrees		Session (_): Date:<br>Phase + Dosage:
**Phase 3**	Standing Hip Abduction Cables		
	Progress:<br>- External load	Alternatively gold/silver theraband can provide sufficient resistance	Session (_): Date:<br>Phase + Dosage:
**Dosage**<br>**3 x week**	3 x 8-10 (3s up, 1s hold, 3s down) each side to 30 degrees (swap 0s rest)<br>Load: BW or theraband to fatigue at 10 reps/10RM	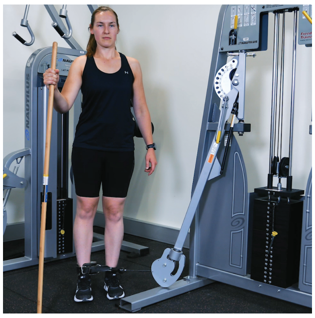<br><br>*Light upper limb support allowed*	Session (_): Date:<br>Phase + Dosage:<br><br>Session (_): Date:<br>Phase + Dosage:

**Pass once completed set dosage + correct technique/functional alignment/area of fatigue, nil response of pain (<2/10) or swelling.
**If struggling with activation, give as 7x week activation work (i.e. pillow between legs). Adjust accordingly in Physitrack 7x week for this exercise

**TASK 5: Calf**		Description of Exercise	Physiotherapy Log
**Phase 1**	"Calf Raise" Bilat - BW Double Leg<br>"Soleus Raise" – BW Double Leg	*Emphasise medial calf /foot intrinsic/Pushing through big toe*<br><br>*Focus on technique, foot control*<br><br>*Note focus on patient deficits and bias knee extended vs knee flexed as appropriate*<br><br>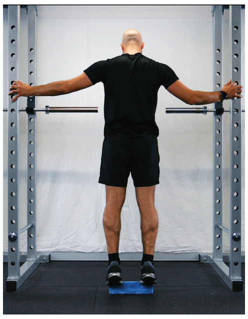	Session (_): Date:<br>Phase + Dosage:
**Dosage**	3 x 30 (2s up 1s hold 2s down) (Rest 30s) Full available ROM<br><br>5 x week<br>3 sessions: KE<br>2 sessions: KF (or vice versa depending on patient)		Session (_): Date:<br>Phase + Dosage:
Progress to Phase 2*	3 x 20 body weight correct fatigue area and form		
**Phase 2**	"SL Calf Raise" - Body Weight<br>"SL Soleus Raise" – Body Weight		Session (_): Date:<br>Phase + Dosage:
**Dosage**	3 x 15 - 30 (2s up 1s hold 2s down) (Rest 30s) Full available ROM<br><br>3 x week /every 2nd day<br>2 sessions: KE<br>1 sessions: KF (or vice versa depending on patient)		Session (_): Date:<br>Phase + Dosage:
Progress to Phase 3*	1. 3 x 25 BW<br>2. >95% LSI (max reps compared side to side)		
**Phase 3**	"SL Calf Raise - off Step" (Body Weight)<br>"SL Soleus Raise - off Step" (Body Weight)	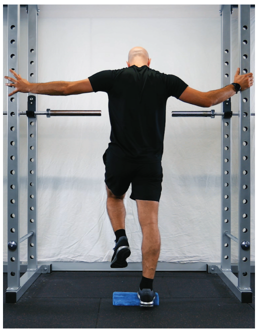	Session (_): Date:<br>Phase + Dosage:
**Dosage**<br>**3 x week**	3 x 10-12 (2s up 1s hold 2s down) (Rest 30s)<br><br>3 x week/every 2nd day<br>2 sessions: KE<br>1 sessions: KF (or vice versa depending on patient)		Session (_): Date:<br>Phase + Dosage:
Progress to Phase 4*	1. 3 x 25 BW<br>2. >95% LSI (max reps compared side to side)		Session (_): Date:<br>Phase + Dosage:
**Phase 4**	"SL Calf Raise off Step" with external load<br>"SL Soleus Raise off Step" with external load	Note: option for Smith Machine Seated for soleus<br><br>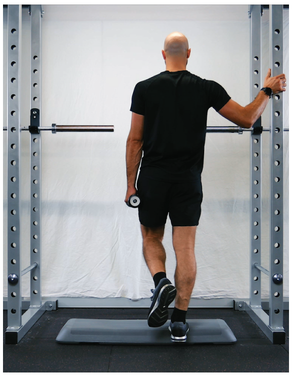	
**Dosage**	3 x 12 (2s up, 1s hold, 2s down) (Rest 30s) Full available ROM<br><br>3 x week /every second day<br>2 sessions: KE<br>1 sessions: KF (or vice versa depending on patient)		Session (_): Date:<br>Phase + Dosage:
Ongoing progressions	Continue to build load to maintain dosage 3 8-12RM		

*Pass once completed set dosage + correct technique/functional alignment/area of fatigue, nil response of pain (<2/10) or swelling.

**TASK 6: TRUNK^**		Physiotherapy Log
**Phase 1**	Bench Plank – 45 degrees – 30 degrees	Session (_): Date:<br>Phase + Dosage:
	Progress:<br>- Height of bench (45 – 30 – 15 degrees) to elbows on ground (0 degrees body angle)	
**Dosage**	3 x 60s isometric hold (60s rest)<br>3 x week/every 2nd day	
Progress to Phase 2*	60s elbows on ground /0 degrees body angle with good trunk control	Session (_): Date:<br>Phase + Dosage:
**Phase 2**	"Elbow Plank"	
	Progress:<br>- Push Up Plank<br>- Push Up Plank with hip knee drive (+/-)theraband	Session (_): Date:<br>Phase + Dosage:
**Dosage**	3 x 60s isometric hold (60s rest)<br>3 x week/every 2nd day<br>Knee drive – 3 x 10 reps each side (2s out 2s in)	Session (_): Date:<br>Phase + Dosage:
Progress to Phase 2*	60s on knee drive with theraband	
**Phase 3**	"Side plank series" (start on knees)	
	Progress:<br>- On toes and elbow<br>- On toes and hand	Session (_): Date:<br>Phase + Dosage:
**Dosage**	3 x 60s isometric hold (60s rest)<br>3 x week/every 2nd day	
Progress to Phase 2*	60s on toes with arm and leg raise	Session (_): Date:<br>Phase + Dosage:
**Phase 4**	Rotational trunk series<br>- Palov press or rotations with theraband or cable<br>- Side plank with rotation with weight<br>Other Physitrack options – cable woodchop	Session (_): Date:<br>Phase + Dosage:
**Dosage**	3 x 12 reps (2s out, 2s in) (60s rest)<br>3 x week/every 2nd day	
Ongoing Progressions*	Continue to challenge patient as appropriate or specific to their sport.	Session (_): Date:<br>Phase + Dosage:

**Pass once completed set dosage + correct technique/functional alignment/area of fatigue, nil response of pain (<2/10) or swelling.

Key points as per video:

- *Keep pelvis tucked/squeeze gluts*
- *Sink shoulder blades*
- *Neck neutral*
- *Reinforce into functional movements and activities*

TASK 7: Gluteal/Hamstring

**Phase 1**	Double Leg Bridge
**Dosage**	Activation: 3 x 15 reps (2s up, 2s down) + 20s hold (30s rest)<br>7 x week#
Progress to Phase 2*	Feeling gluteals working for 3 x 20 DL<br>Feels gluteals > hamstring working in SL trial
**Phase 2**	Single Leg bridge<br>Progress:<br>- Assisted to unassisted
**Dosage**	3 x 15 reps (2s up, 2s hold 2s down) (60s rest)<br>3 x week/ every 2nd day
Progress to Phase 3*	Full height:<br>1. >95% LSI<br>2. > 15 reps max<br>3. Fatigue - mainly gluteals
**Phase 3**	Hamstring Bridge series (on bench)<br>Progress:<br>Double – single
**Dosage**	3 x 15 (2s up, 2s down) (60s rest)<br>3 x week / every 2nd day
Progress to Phase 4*	3 x 15 single leg
**Phase 4**	Hamstring Ball Curl series<br>Progress:Double – single<br>Combine with DL- do 3 SL and 9 DL
**Dosage**	3 x 12 (2s out, 0 hold 2s in) (60s rest)<br>3 x week / every 2nd day
**Phase 5**	Nordics<br>Progress:<br>Assisted (theraband) – unassisted
**Dosage**	3 x 4-6 (2s out, hold 2, 2s in)<br>3 x week / every 2nd day
Ongoing Progressions*	To physio discretion according to patient needs

Physiotherapy Log

Session (_): Date:
Phase + Dosage:

Session (_): Date:
Phase + Dosage:

Session (_): Date:
Phase + Dosage:

Session (_): Date:
Phase + Dosage:

Session (_): Date:
Phase + Dosage:

Session (_): Date:
Phase + Dosage:

Session (_): Date:
Phase + Dosage:

Session (_): Date:
Phase + Dosage:

*Pass once completed set dosage + correct technique/functional alignment/area of fatigue (as per video), nil response of pain (<2/10) or swelling.

#In Physitrack still delete Days 2, 4, 6, 7 still, then "Add exercise" go to exercise and click add to specific "off" days usually 2,4,6,7.

<u>TASK 8: Sport Specific/Cardiovascular^</u>		Description of Exercise	Physiotherapy Log
**Phase 1**	**Non-impact cross training**	Walking<br>Bike<br>Swimming<br>Cross Trainer<br>Deep water jogging<br>Rowing<br><br>PLUS Non-impact Sport Specific Skills	Session (_):<br>Date:<br>Phase + Dosage:<br><br>Session (_):<br>Date:<br>Phase + Dosage:
	Phase 1.1 10 mins		
	Phase 1.2 20 mins		
	Phase 1.3 30 mins		
	Phase 1.4 30 mins (inc 5x60s high intensity, 90s rest)		
	Phase 1.5 30 mins (inc 5x2mins high intensity, 120s rest)		
	Phase 1.6 45 mins with 15 mins high intensity		
Progress to Phase 2*	Return to Running Outcome Measures (See section 4.6)		
**Phase 2***	**Running + Agility Program**	Note: distance/time can be catered to individual needs/sport<br>At least one rest day between sessions<br><br>**Running progressions:**<br>1. Walk: jog (5:1, 4:2, 3:3, 2:4, 1:5)<br>2. Continuous<br><br>**High intensity progressions**<br>Total time (10-20 minutes) can be made up of:<br>1. Straight line efforts<br>2. Straight line acceleration/deceleration<br>3. Agility program (example attached)<br><br>*Straight line efforts*<br>Moderate speed i.e. 14 – 16km/hr or 75%<br>High speed i.e. 16 – 20km/hr or 80-90%<br>Sprint i.e. 20-23km/hr or 100%<br><br>Progress through levels Once completed 2 sessions at 100%move to next level. (120s rest btw sets)<br><br>Level 1: 2 x 200m on 90 secs, Repeat x 2 sets<br>Level 2: 3 x 200m on 90 secs, Repeat x 2 sets<br>Level 3: 3 x 200m, on 90 secs, Repeat x 3 sets<br><br>*Acceleration/Deceleration:*<br>As above with 2 x 40m spurts of 100% (accel at cone, decel at cone)<br>□ □40m 100%□ □40m 100%□ □<br>200m<br>Level 4: Complete Level 3, then 2 x 200m on 90secs with accel/decel, x 2 sets<br>Level 5: Complete Level 3, then 3 x 300m, on 90secs with accel/decel, x 2 sets<br>Level 6: Complete Level 3, then 3 x 300m, on 90secs with accel/decel, x 3 sets<br>Level 7: Complete as above, but with backpedal x 2 (3rd cone to 2nd cone, then 5th to 4th)<br>(120s rest btw sets)<br><br>***Agility*** **-** See attached section: Note must have completed straight-line accel/decel Level 4 before can start 70% agility runs.	Session (_):<br>Date:<br>Phase + Dosage:<br><br>Session (_):<br>Date:<br>Phase + Dosage:<br><br>Session (_):<br>Date:<br>Phase + Dosage:<br><br>Session (_):<br>Date:<br>Phase + Dosage:<br><br>Session (_):<br>Date:<br>Phase + Dosage:<br><br>Session (_):<br>Date:<br>Phase + Dosage:
	Phase 2.1 Run 15 minutes + 30 mins Phase 1		
	Phase 2.2 Run 20 minutes + 25 mins Phase 1		
	Phase 2.3 Run 30 minutes + 20 mins Phase 1		
	Phase 2.4 Run 45 minutes inc 10 mins high intensity/agility		
	Phase 2.5 Run 50 minutes inc 20 mins high intensity/agility		
Progress to Phase 3*	>90% Return to Sport Score		
**Phase 3**	**Non-Contact Training**		
	Phase 3.1 Sport 15 minutes + 30 mins Phase 2 running		
	Phase 3.2 Sport 20 minutes + 25 mins Phase 2 running		
	Phase 3.3 Sport 30 minutes + 20 mins Phase 2 running		
	Phase 3.4 Sport 45 minutes including 15 mins high intensity running /agility		
	Phase 3.5 Sport 50 minutes including 20 mins high intensity running + 10 minutes low intensity running		
Progress to Phase 4*	>95% RETURN TO SPORT SCORE		
**Phase 4**	**Full training**		
	Full training up to 1 hour 3 x week (no match play)		
	Full training up to 1.5 hour 3 x week (no match play)		
	Full training including match play up to 4 x week		
Progress to Phase 5*	Dosage, nil adverse effects		
**Phase 5**	**Return to match play**		
	Match play 45-60 minutes x 1 week		
	Full match load 60 minutes + 1 x week		
	Monitor for adverse effects		

*Pass once completed set dosage + correct technique/functional alignment/area of fatigue, nil response of pain (<2/10) or swelling.

ON PHYSITRACK – add the type of CVS exercise I.e. bike or running then add manually what running etc they are doing

TREATMENT GUIDE FOR TIER 1 – PASSIVE THERAPY

Target for treatment	Technique	Aim	Description	Dosage	Home Exercises	Timeline
Overactive secondary stabilisers	Soft tissue massage and trigger point release of pes anserine group, adductor group, vastus lateralis, rectus femoris, popletius, gastrocnemius group, hamstring group, gluteal/ITB	Address soft tissue restrictions with the aim of:<br>Reducing pain<br>Increasing knee joint range of movement<br>Improving muscle activation<br>Improving symptoms during function	Sustained digital pressure to each trigger point with the muscle positioned on stretch<br>OR<br>Massage longitudinally along the muscle belly	30-60 seconds digital pressure per trigger point<br><br>2-5 minutes of massage per muscle	Self release/trigger point/stretching of appropriate muscle group<br>Foam Roller – 2 x 10 each muscle group. 2-3 x daily<br>Spikey Ball- 3 x 30s each point. 2-3 x daily<br>Stretching – 3 x 30s each group 2-3 x daily	As required/until passed Tier 1 criteria to enter Tier 2 Rehab Program. See Assessment Log Book for guidance.
Swelling/ Effusion	Patient education	Determine aggravating factors<br>Educate about management of aggravating and addressing contributing factors	Education: Swelling vs joint effusion, inflammatory factors and cartilage<br>Contributing factors to joint load	NA	Reducing aggravating factors to allow joint to settle<br>Address contributing factors (range, strength, biomechanics)	
"PFJ"<br>Anterior knee pain or stiffness felt on palpation	PFJ mobilisation techniques<br><br>Taping techniques	Address PFJ mobility restrictions to improve knee joint range of movement, pain, muscle activation<br>Alleviate PFJ contact pressure with taping, reduction of aggravation factors	Lateral-medial glides, grade 111 or IV in position of restriction i.e. sidelying 30 degrees<br>Taping: rigid, kinesiology techniques	3-5 sets of 30-60 seconds	Self mobilisation/release of appropriate soft tissue structures<br>Self taping<br>Reducing aggravating factors/addressing contributing factors as above	
ROM	Various: Inc knee extension/flexion range of motion by addressing appropriate contributing factor:	Bony end feel<br><br>Soft tissue soft end feel/muscle protective	Treat with respect<br><br>STW as per above OR<br>Hold relax techniques OR<br>Gr II or IV physiological mobilisation	NA<br><br>2-5 minutes<br>3-5 sets, 30s hold<br>3-5 sets of 30-60s	Range of motion exercises at discretion of physiotherapist<br>- AROM 5 daily x 20<br>- Prone Leg hang<br>Self release if muscle protective	
Muscle weakness<br>-Quad<br>- Glut<br>- Calf<br>- Hamstring	Activation techniques	Improve activation through hands on feedback, taping	Therapist and patient hands on touch to muscle activation in sitting, bridging exercises<br>Kinesiology active taping	2-5 minutes	- Quads activation/STS practice 3 x 20 (DL –SL)<br>- Bridging 3 x 20 (DL-SL)<br>- Calf Raises 3 x 20 (DL-SL)<br>- SL Stance 3 x 30s (EO-EC)	
Gait	Re-education techniques	Determine any biomechanical deficiencies. I.e. Dec KE stance, Dec KF swing. Assess in part practice, compare to other side	Education about gait deficiencies and how leads to increased PFJ pressure and break down components<br>Video/mirror feedback/part practice	2-5 minutes	Gait part practice – whole practice. I.e. stance with theraband / inner range quads control. Progress to step up. 3 x 8	

AGILITY PROGRAM – on physitrack

Distance can be varied according to individual sport/fitness level

Drill	Angle Progressions	Speed % Max	Dosage
Phase 1 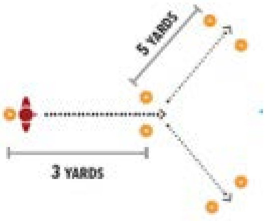	45 degrees	70<br>80<br>90<br>100	3 X 5<br>3 X 4<br>3 X 3<br>3 X 2
Phase 2 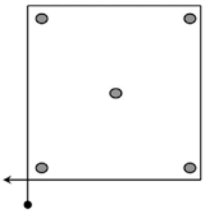	90 degrees	70<br>80<br>90<br>100	3 X 5<br>3 X 4<br>3 X 3<br>3 X 2<br><br>+/- 2 x 2 minutes skipping/ladder
Phase 3 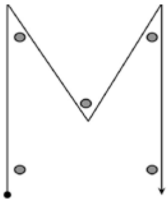	135 degrees	70<br>80<br>90<br>100	3 X 5<br>3 X 4<br>3 X 3<br>3 X 2<br><br>+/- 2 x 2 minutes skipping/ladder
Phase 4 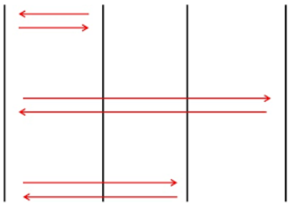	180 degrees	70<br>80<br>90<br>100	3 X 5<br>3 X 4<br>3 X 3<br>3 X 2<br><br>+/- 2 x 2 minutes skipping/ladder
Phase 5<br>- Walking grapevine 20m<br>- Increased speed grapvine 20m<br>- "S" bends<br>- Run backwards 10m, turn and go R or L (anticipated – unanticipated)	Pivoting/twisting	70<br>80<br>90<br>100	X 6
Phase 6<br>Multidirectional & unanticipated<br>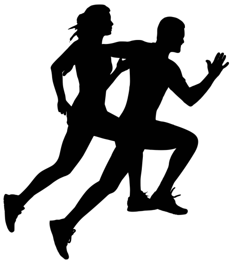 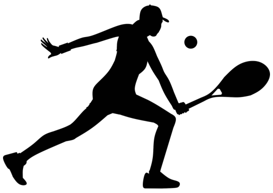	- Reaction ball Partner mirroring<br>- Partner skill work non contact i.e. basketball defensive slides against ball carrier<br>- Partner skill work contact i.e. basketball boxing out	70<br>80<br>90<br>100	10 - 20 minutes<br><br>Integrate into individual skill and partner drills

PHYSIOTHERAPY PROGRAM: TASK PROJECT (Group: Devil) Participant ID:

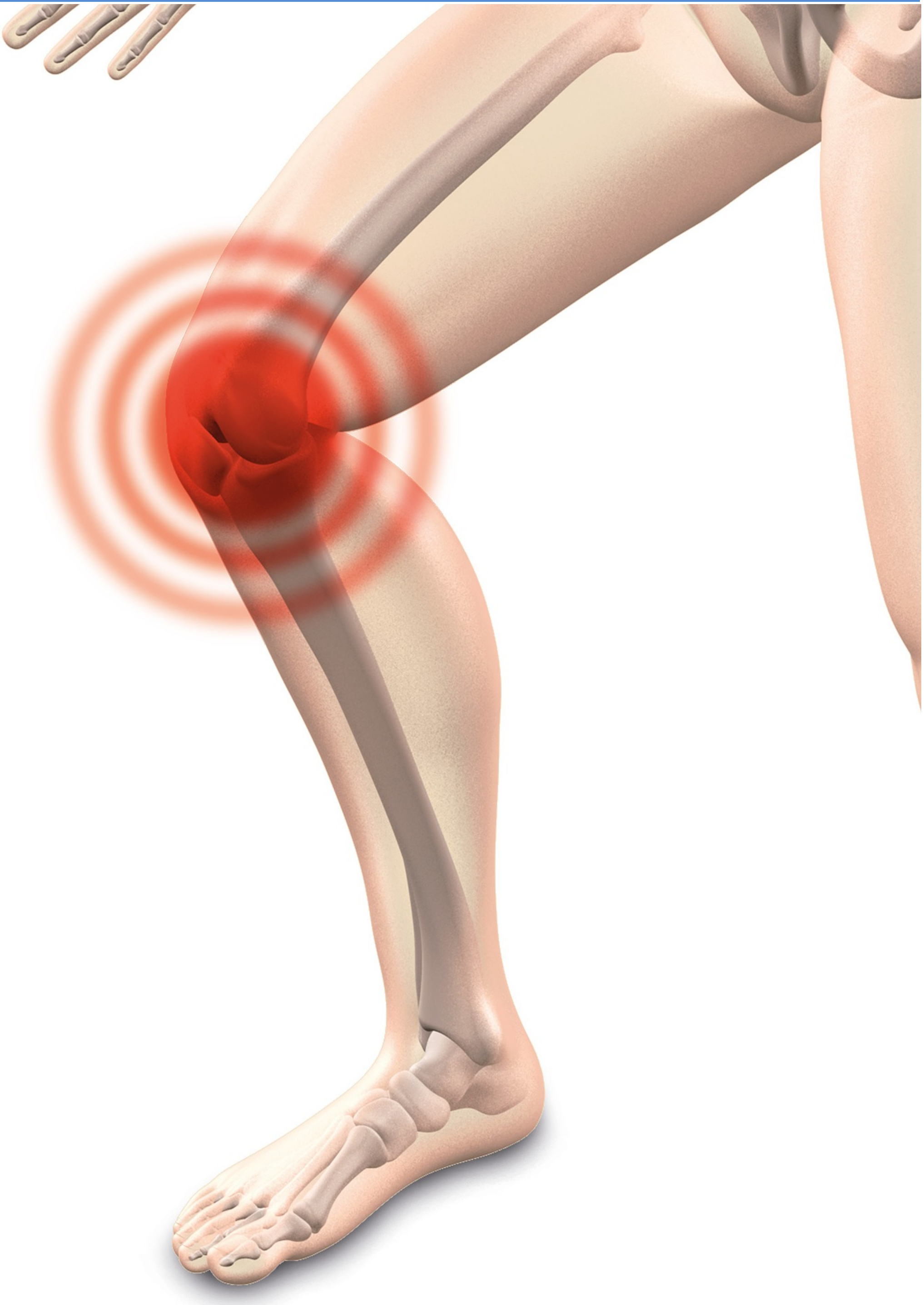

This booklet contains:

- Trunk strengthening exercise therapy protocol
- Physiotherapist clinical notes and attendance record
- Educational resources and handouts
- **Other resources you may need:**
- Administrative procedures physiotherapy handbook

OVERVIEW OF PROGRAM

General overview
The control group intervention consists of standardised trunk strengthening, stretching and education.

Patients are to attend physiotherapy appointments as per the intervention group (8 sessions of 30 minutes duration

Whilst this is a "control group" we ask that you deliver this intervention with equal enthusiasm as the targeted ACL-specific protocol, and educate the participants on the value of trunk strength and lumbo-pelvic control for lower limb neuromuscular function, biomechanics and sport performance.

- **Trunk strengthening**
- **Stretching**
- **Education**

1. Exercise-therapy protocol
Choose a minimum of three trunk exercises. Progress each patient according to their level of ability through the trunk strengthening exercises. They require minimal to no equipment. (Page 16)

2. Stretching
Lower limb and lumbo-thoracic stretches and mobility appropriate for the patient should be prescribed (Page 17)

Participants are encouraged to perform the exercises independently 3 times per week, and record their adherence via Physiapp.

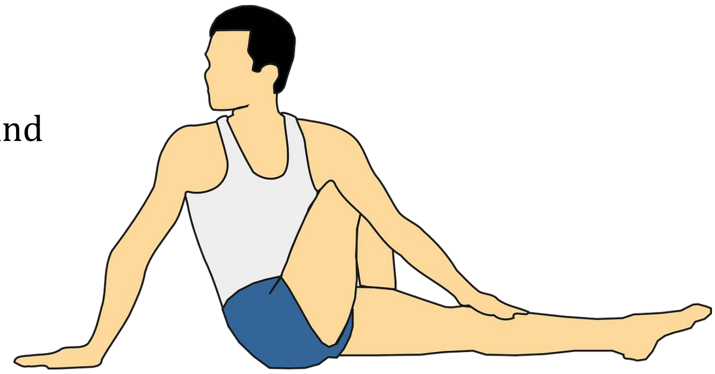

3. Education
Education topics for the control group are outlined on Page 20. As the control group contains only trunk strength, patients are not educated on the importance of strengthening other muscle groups and specific return to sport criteria. However, if wishing to return to sport they are encouraged to receive advice and clearance from their surgeon.

<table>
<tr><th colspan="2"><u>TRUNK STRENGTH</u></th><th>Description of Exercise Program</th><th>Physiotherapy Log</th></tr>
<tr><td rowspan="2">Exercise 1</td><td>“Active alternate UL and LL extensions over ball”
- Or in “4 point kneeling”</td><td rowspan="12">Even though this is the control group ensure remain positive about the effectiveness of trunk strengthening, education and stretching program

First 5 sessions use exercises 1 E 3 Big appropriate difficulty of that exercise for patient
Make sure they get a good work out within the session – perform 3 sets of each of the 3 exercises.

Education re trunk and pelvic position and relationship to muscle function and lower limb biomechanics
Demonstrate pelvic position in lying and standing
+ Patient practice

Education re exercise program:
- Progress as able to achieve technique and dosage
- Minimum 3 x week</td><td rowspan="19">Session (_): Date:
Phase:
Assessment:

Session (_): Date:
Phase:
Assessment:

Session (_): Date:
Phase:
Assessment:

Session (_): Date:
Phase:
Assessment:

Session (_): Date:
Phase:
Assessment:

Session (_): Date:
Phase:
Assessment:

Session (_): Date:
Phase:
Assessment:

Session (_): Date:
Phase:
Assessment</td></tr>
<tr><td>Arm Lift – Leg Lift – Arm and Leg Lift
Alternate/fun challenge in session: Both Arms and Legs</td></tr>
<tr><td>Dosage</td><td>3 x 30 - 60 seconds</td></tr>
<tr><td>PASS</td><td>Dosage with good control Arm & Leg Lift</td></tr>
<tr><td>Exercise 2</td><td>“Dead bugs”
Toe taps – leg extension</td></tr>
<tr><td></td><td>Alternate/fun challenge in session: ball between legs and pass to hands</td></tr>
<tr><td>Dosage</td><td>3 x 30 - 60 seconds</td></tr>
<tr><td>PASS</td><td>Dosage with good control on toes</td></tr>
<tr><td rowspan="2">Exercise 3</td><td>Plank series
- “30 degree bench plank”
- “Elbow plank”
- “Plank in push up position”
- “Plank – one arm row”</td></tr>
<tr><td>Alternate/fun challenge: Ball roll outs</td></tr>
<tr><td>Dosage</td><td>3 x 30 - 60 seconds</td></tr>
<tr><td>PASS</td><td>Dosage with good control</td></tr>
<tr><td>Exercise 4</td><td>“Anti rotation series in standing”
- Palov press
- Rotation with theraband then cable</td><td rowspan="3"></td></tr>
<tr><td>Dosage</td><td>3 x 30 - 60 seconds</td></tr>
<tr><td>PASS</td><td>Dosage with good control</td></tr>
<tr><td rowspan="2">Exercise 5</td><td>Free choice trunk exercise</td><td rowspan="4"></td></tr>
<tr><td>According to patient needs</td></tr>
<tr><td>Dosage</td><td>3 x 30 - 60 seconds</td></tr>
<tr><td>PASS</td><td>Dosage with good control</td></tr>
</table>

Stretching and mobility

- *Education to patient - to improve joint and muscle mobility*
- *Choose most appropriate for patient*

	**Options (type name into physitrack)**	**Dosage**	**Description**
Physio to circle most appropriate for individual patient	1. Side-lying thoracic rotations<br>2. Thoracic foam roll<br>3. Quadriceps stretch<br>4. Hip flexor stretch<br>5. Hamstring stretch<br>6. Gluteal myofascial release with ball<br>7. Rotational spine stretch<br>8. Calf stretching standing (gastrocs)<br>9. Soleus stretching	As appropriate to patient	See videos on Physitrack

The following pages education leaflets are to be provided to patients in the lower-limb focussed group

1. **What does the surgery involve?**
2. **Rehabilitation & Return to Sport**
3. **Can I prevent future injury?**
4. **Psychological Impact of Injury**
5. **Things to look out for**

The following pages education leaflets can be provided to participants in the trunk-focussed group

1. **What does the surgery involve?**

4. **Psychological Impact of Injury**
5. **Things to look out for**

1. What does the surgery involve?

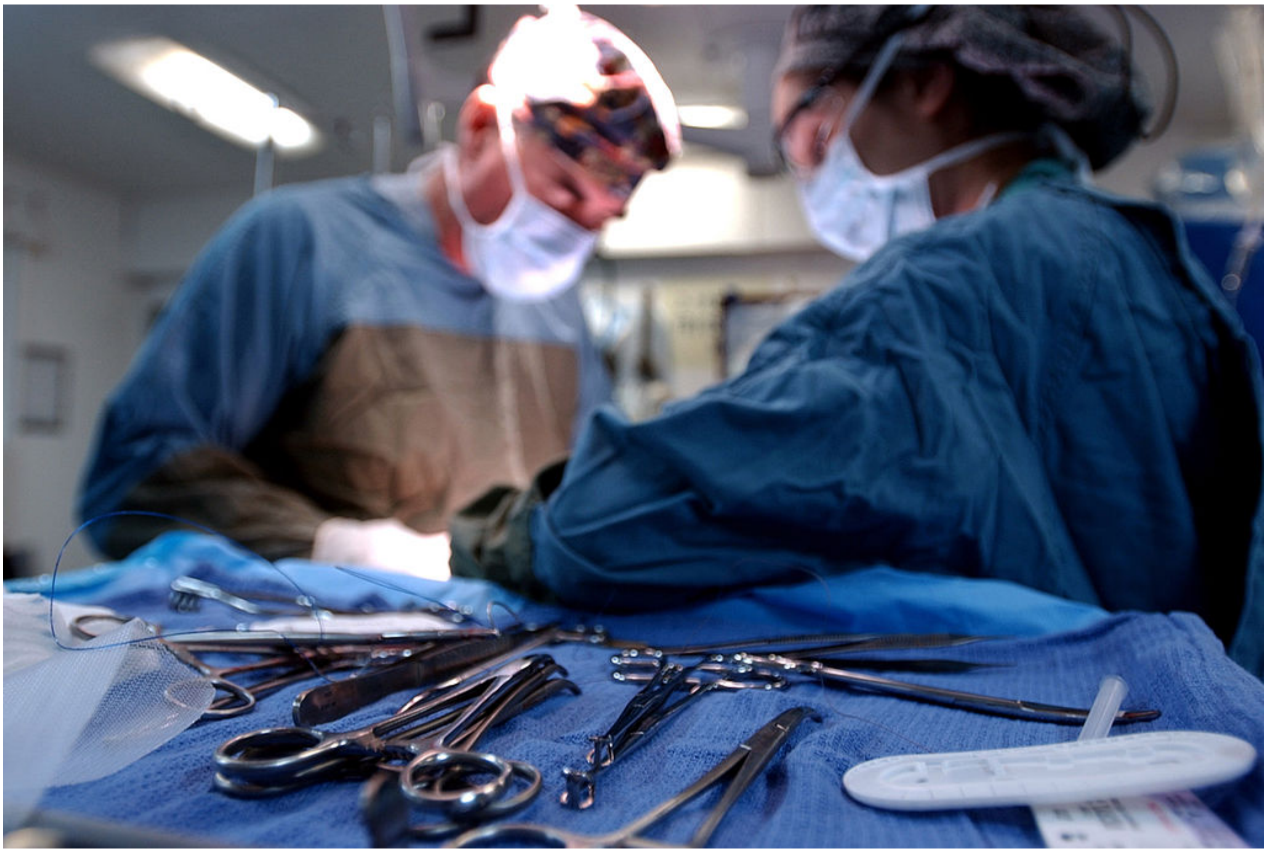

This education leaflet is designed to give you a brief and basic overview of anterior cruciate ligament reconstructive surgery

Every patient, injury and surgeon is different therefore please consult your health professional for further individual detail

Further information related to this topic can be found at:

semrc.blogs.latrobe.edu.au

For a great visual summary of ACL reconstructive surgery go to the following YouTube® video "ACL Surgery – 3D Reconstruction"

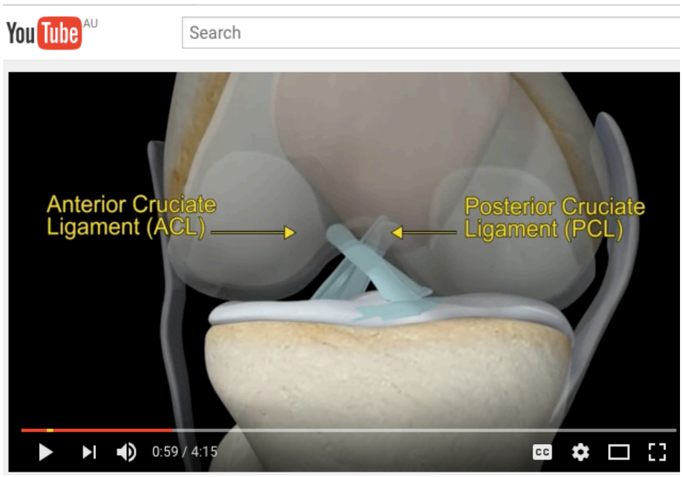

LA TROBE UNIVERSITY

PATIENT EDUCATION
ANTERIOR CRUCIATE LIGAMENT RECONSTRUCTION

Main Graft Types

Autograft

- Tendon comes from you (hamstring or patella tendon)
- Risk of rejection is low due to tissue being native (from you)

Preference

- Surgeon/patient dependent but outcomes are similar regardless
- E.g. patella may be preferred if previous hamstring injuries

Hamstring and Patella Tendon Grafts

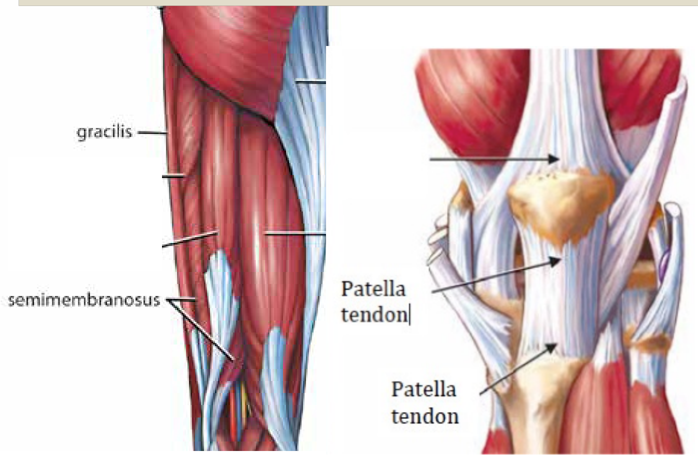

Back of knee　　Front of knee

What to expect after surgery

It is normal to experience in the first few weeks:

- Pain, swelling, muscle wastage, stiffness

You will be given advice about:

- Use of crutches
- Pain medication
- Ice, elevation and compression
- Gentle exercise vs rest

Other injuries

The difference between meniscus and cartilage

"*Cartilage*" refers to the "articular cartilage," the smooth protective covering over the bone

The "*meniscus*" are different types of shock absorbing cartilage. They are the "C" shaped discs that sit inside the knee.

Injuries to the cartilage and/or the meniscus commonly occur at the same time as injury to the ACL.

Other surgeries

Repair vs resection

Damage to cartilage or meniscus may be repaired using a variety of fixation methods (stiches/anchors). If this is not possible it may be left, or taken out/"resected"

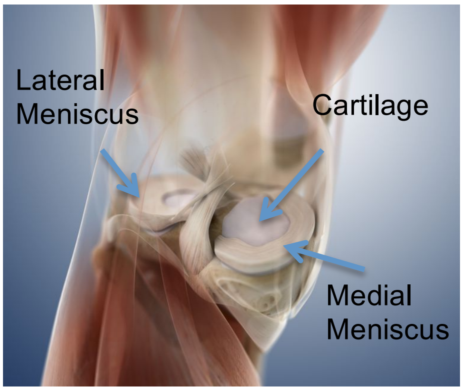

Further information

Website/Blog
semrc.blogs.latrobe.edu.au/category/acl

Facebook
facebook.com/latrobesemrc

Twitter
twitter.com/LaTrobeSEM

2. Rehabilitation & Return to Sport

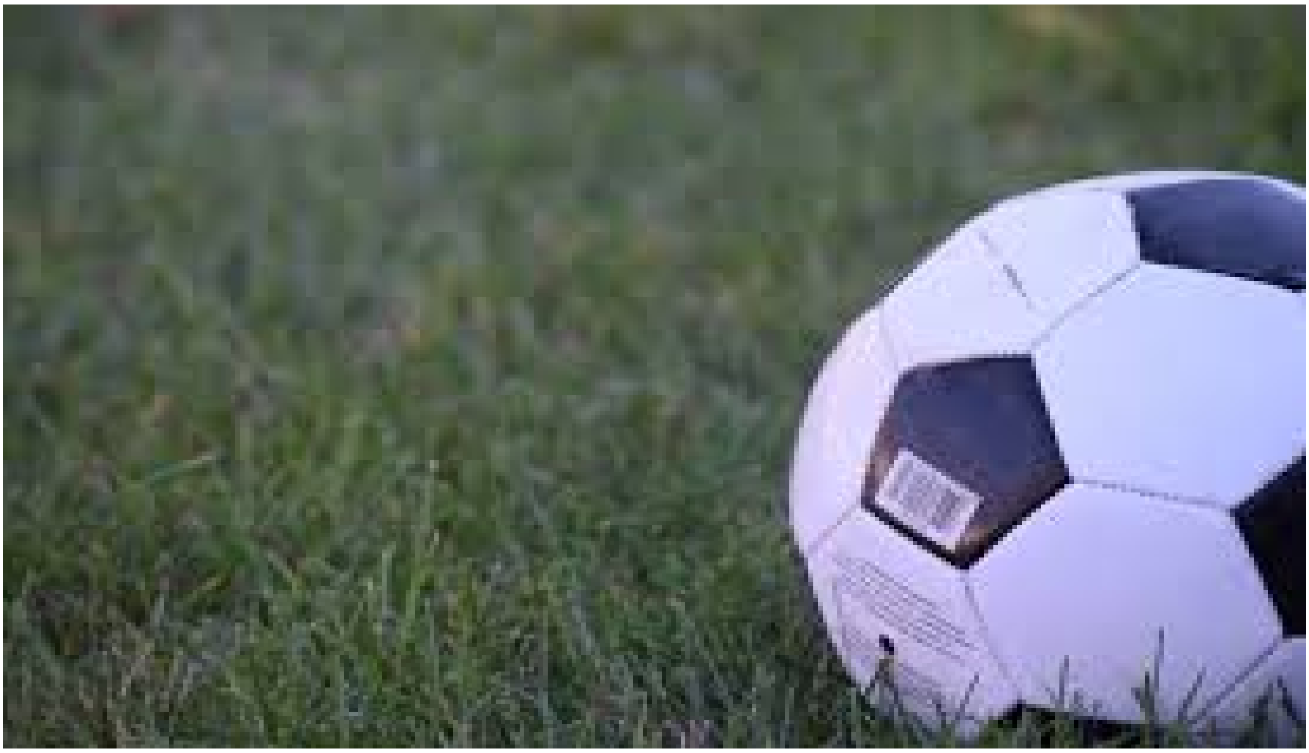

This education leaflet is designed to give you a brief and basic overview of the rehabilitation and return to sport process following anterior cruciate ligament reconstruction

Every patient, injury and surgeon is different therefore please consult your health professional for further individual detail

Further information related to this topic can be found at: semrc.latrobe.edu.au

Rehabilitation following anterior cruciate ligament reconstruction is challenging and at times you may wish to discontinue for a variety of reasons (cost, time, ability to self manage, decreased motivation)

You should continue formal rehabilitation with a health professional until your operated leg is as strong as your other leg (if not stronger) to reduce the risk of re-injury and possibly future joint health

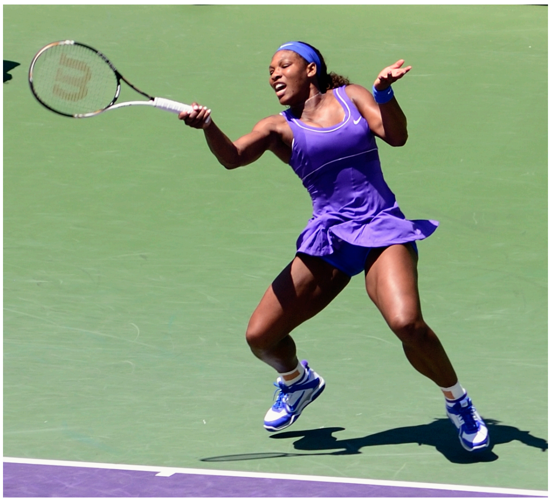

PATIENT EDUCATION
ANTERIOR CRUCIATE LIGAMENT RECONSTRUCTION

LA TROBE UNIVERSITY

Return to Sport Criteria

Returning to sport with ongoing strength and movement deficits may place you at risk of abnormal joint/muscle loading, or reinjury. It is therefore important to continue to see your health professional for assessment and treatment of strength/movement deficits.

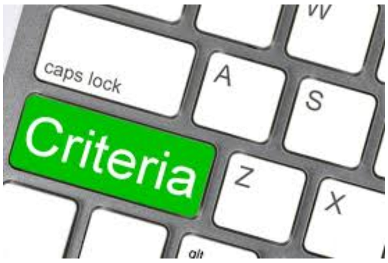

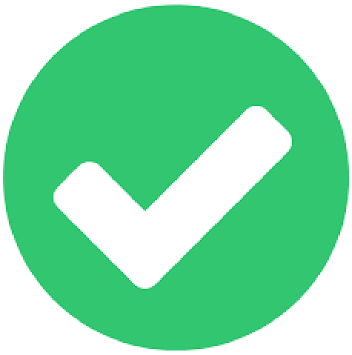

1. Nil to minimal **pain** and **swelling**
2. **Full range of motion**
3. **“Full” strength** of all muscle groups (= >95% of opposite side & >values for non injured people). This includes calf, quadriceps, hamstring, gluteals and trunk muscles.
4. **“Restored” function and movement.** I.e. walking, running, jumping, landing/cutting. This is not only how far you can run or hop but normal “movement patterns”
5. **Completed a graded return to activity.** I.e. - cardiovascular exercise non-impact exercise – running – speed/agility –modified – full training – match simulation – return to games. No more than 10% increase each week
6. **Psychological readiness** (see leaflet no. 4)

Your physiotherapist will assist you in assessing these items formally. Achieving all of these criteria typically takes 12 months, however it can take up to 2 years.

What if I do not want to return to sport?

- This is a completely justified decision, however restoring full symmetry in strength and function is still important for future joint health.
- Restoring all movements is also important as it may required in a social setting (i.e. playing sport with family/children)

Further information

Website/Blog
semrc.blogs.latrobe.edu.au/category/acl

Facebook
facebook.com/latrobesemrc

Twitter
twitter.com/LaTrobeSEM

3. Can I prevent future injury?

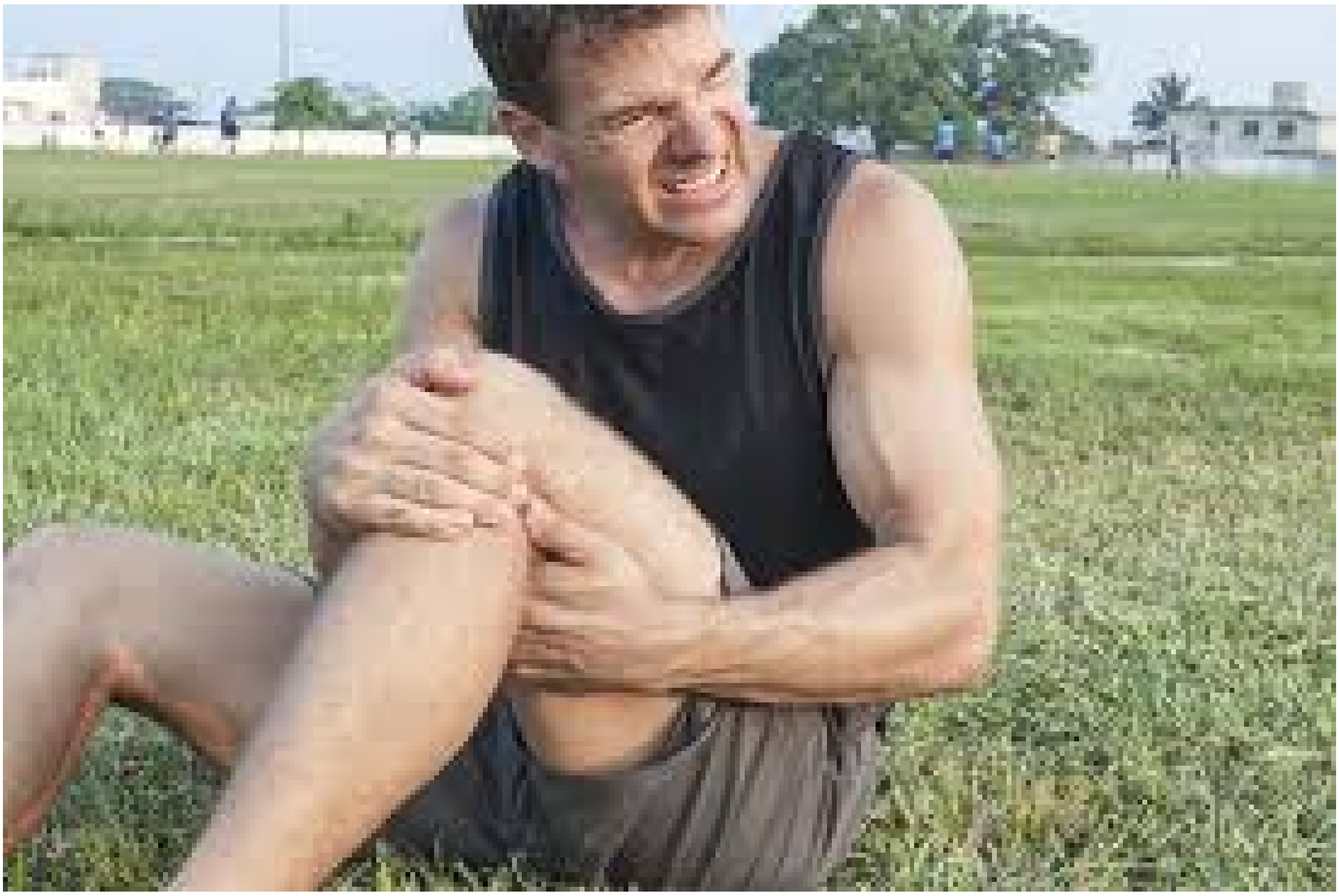

This education leaflet is designed to give you a brief and basic overview of injury prevention following anterior cruciate ligament reconstruction

Injury prevention does not just refer to acute injuries or re-rupture of the ACL, injury prevention is important for prevention of meniscus, cartilage wear and tear and all lower limb joint health.

Further information related to this topic can be found at:

semrc.blogs.latrobe.edu.au

Topics covered will include:

- Risk factors for re injury
- What is it and how do I do injury prevention?
- For how long?

PATIENT EDUCATION
ANTERIOR CRUCIATE LIGAMENT RECONSTRUCTION

LA TROBE UNIVERSITY

Risk factors for re-injury

1. Returning to sport too early and not meeting "discharge criteria" prior to return to sport (see leaflet no.2)

2. "Altered movement patterns." People who sustain a second knee injury (re-injury/opposite leg ACL injury) land differently compared to those who do not sustain a second injury.

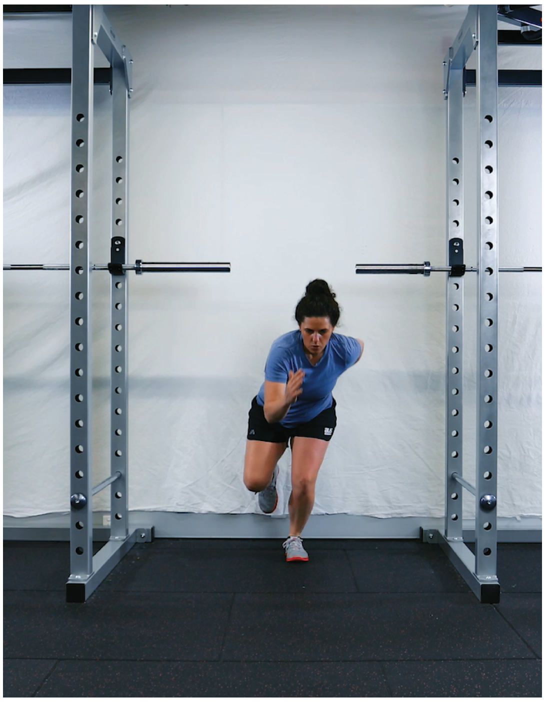

A large part of rehabilitation should include retraining movement patterns – see your health professional

Injury Prevention Key Point 1:

Ongoing Rehabilitation

A large part of injury prevention is maintaining an ongoing strength and conditioning program

2-3 x week to maintain strength & movement patterns in combination with your leisure activities.

The types of exercises you perform may change over your life (see your health professional for program check ups if you have symptoms/functional ability changes)

FOR LIFE?! Strength exercises are important to continue not only for general health but to optimize loads for joint health.

2 – 3 x week in combination with sport/leisure activities

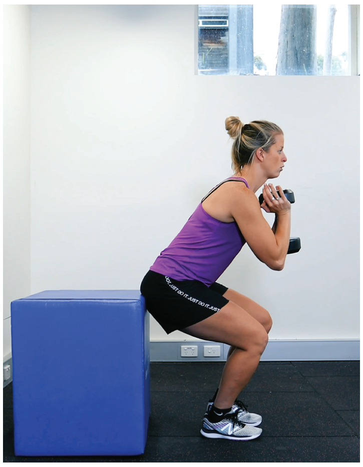

Further information

Website/Blog
semrc.blogs.latrobe.edu.au/category/acl

Facebook
facebook.com/latrobesemrc

Twitter
twitter.com/LaTrobeSEM

Injury Prevention Key Point 2: Injury Prevention Warm Up Programs

As well as performing your regular strength and control exercises, it is important to incorporate an **injury prevention warm up**” to fire up muscles/movement patterns before activity/sport.

Some of these programs have great videos, posters and manuals

1. **PEP program: http://smsmf.org/files/PEP_Program_04122011.pdf**
2. **FIFA 11+ program: http://f-marc.com/11plus/exercises/**
3. **Footy First program: https://footyfirstaustralia.wordpress.com/footyfirst-video-footage/**
4. **Netball Knee program: https://knee.netball.com.au/**
5. **Prep to Play (Australian Rules Football): https://coach.afl/female-football**

Get your sporting club implementing them!

Injury Prevention Key Point #3

A large part of injury prevention also includes managing fatigue and load through your body. Some key things to consider may include:

- **Body weight management**
- **Load management**: Avoid large changes in intensity, type or duration of sport/exercise (no more than 10% increase per week). A simple way to measure this is through “RPE = rating of perceived exertion” x minutes of session. I.e. 7/10 difficult session x 60 minutes = 230. If you did exact session 3 x week. Total load for week = 3 x 230 = 690. Could increase to 760 next week.
- **Eat well** (see health professional if unsure)
- **Sleep well**

LA TROBE UNIVERSITY

PATIENT EDUCATION
ANTERIOR CRUCIATE
LIGAMENT RECONSTRUCTION

4. Psychological Impact of Injury

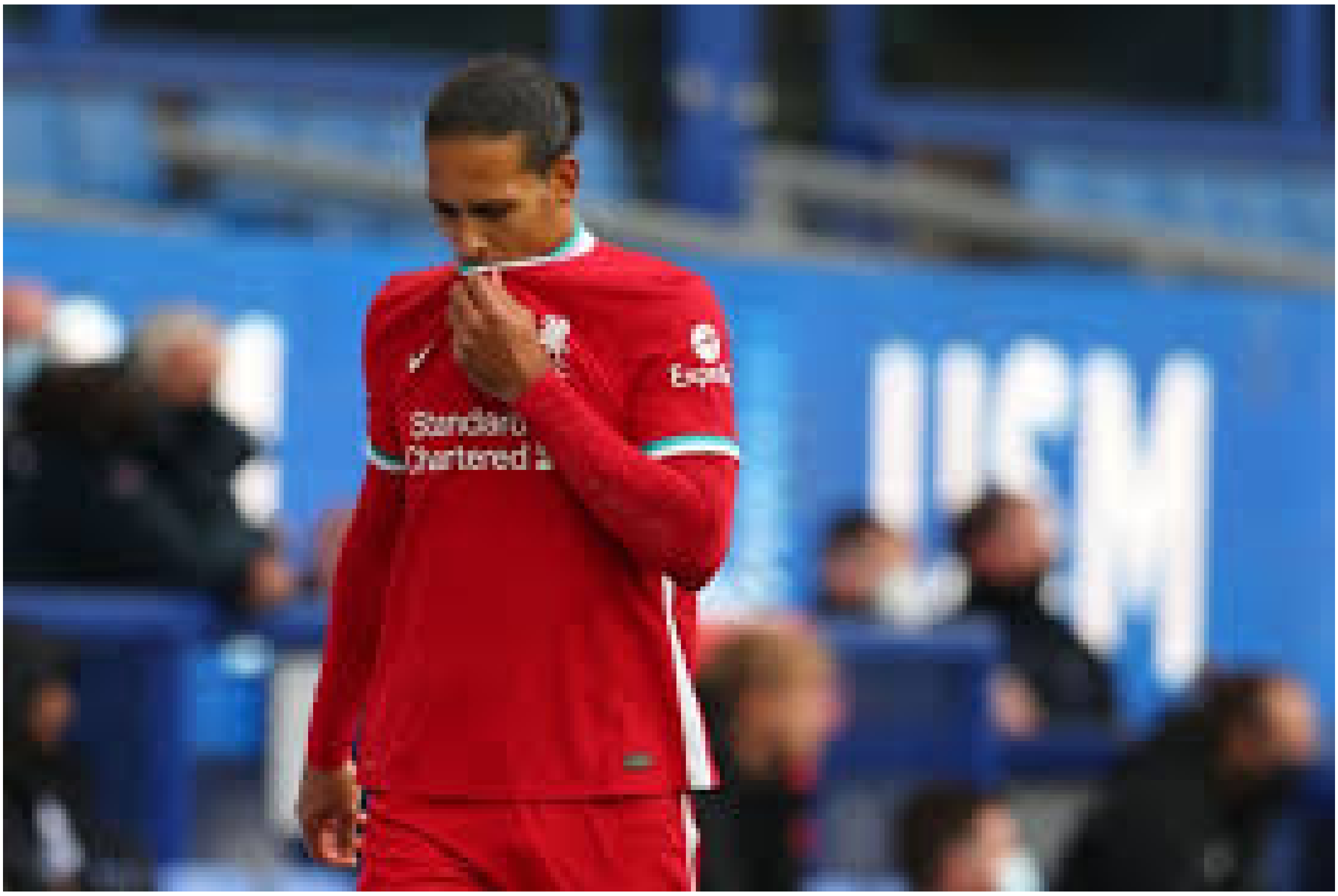

This education leaflet is designed to give you a an insight into the psychological impact of anterior cruciate ligament reconstructive surgery can have

Injury will challenge each individual in different ways and if you are unsure how to best tackle any issues, please consult a qualified health professional

Further information related to this topic can be found at:

semrc.blogs.latrobe.edu.au

Topics covered include:
- Common struggles
- Psychological readiness for sport
- Resources available

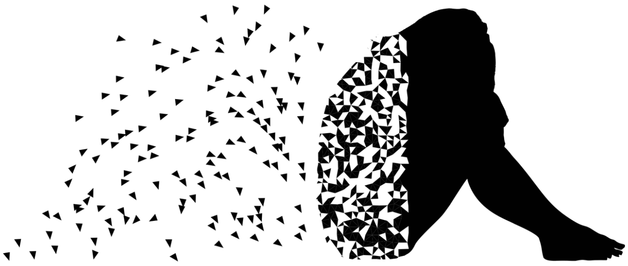

LA TROBE UNIVERSITY

PATIENT EDUCATION
ANTERIOR CRUCIATE LIGAMENT RECONSTRUCTION

Psychological Impact

Fear of re-injury and a lack of trust in your reconstructed knee is very common and you may be having some of the negative thoughts below. However, building strength, and gradually increasing the demands you place on your knee, realistic goal setting, will help you to feel in control.

"It is always in the back of my mind"

"I have changed the way I move and play to reduce the risk of getting injured again"

"I will never be able to ever perform the same"

What can I do about it?

- **Support** from coaches, teammates. Being involved in other ways.
- **Positive** attitude/outlook/mood and motivation toward rehabilitation and return to sport, performance and skills
- Setting **realistic goals** –short and long term with your rehabilitation team along the way is important to aid in **expectations** and prevent dissatisfaction.

Resources you can use:

- Peers that have had an ACL injury
- Your physiotherapist
- Qualified psychologist
- Online website resource created by Daniel Menzel to inspire local sports people: http://www.mtmf.com.au

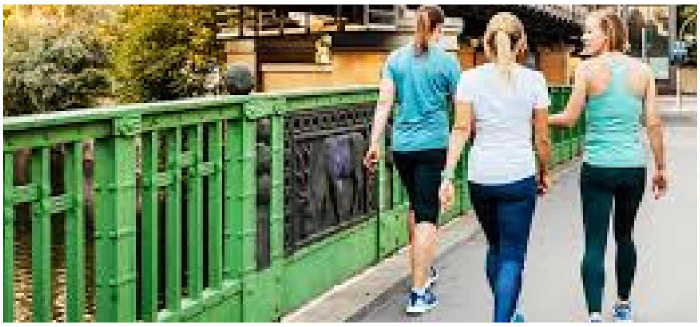

Am I psychologically ready?

1. Prior to return to sport - **physical AND psychological readiness**

2. **ACL specific return to sport after injury questionnaire (ACL – RSI).** Assesses emotions, confidence in performance, and fear of reinjury

See attached copy/smartphone app

People who return to pre injury activity level score significantly higher on the ACL RSI

ACL RSI

Further information

Website/Blog
semrc.blogs.latrobe.edu.au/category/acl

Facebook
facebook.com/latrobesemrc

Twitter
twitter.com/LaTrobeSEM

ACL – RSI (RETURN TO SPORT INDEX)

1. Are you confident that you can perform at your previous level of sport participation?

Not at all confident — Fully confident

☐	☐	☐	☐	☐	☐	☐	☐	☐	☐	☐
0	10	20	30	40	50	60	70	80	90	100

2. Do you think you are likely to re-injury your knee by participating in your sport?

Extremely likely — Not likely at all

☐	☐	☐	☐	☐	☐	☐	☐	☐	☐	☐
0	10	20	30	40	50	60	70	80	90	100

3. Are you nervous about playing your sport?

Extremely nervous — Not nervous at all

☐	☐	☐	☐	☐	☐	☐	☐	☐	☐	☐
0	10	20	30	40	50	60	70	80	90	100

4. Are you confident that your knee will not give way by playing your sport?

Not at all confident — Fully confident

☐	☐	☐	☐	☐	☐	☐	☐	☐	☐	☐
0	10	20	30	40	50	60	70	80	90	100

5. Are you confident that you could play your sport without concern for your knee?

Not at all confident — Fully confident

☐	☐	☐	☐	☐	☐	☐	☐	☐	☐	☐
0	10	20	30	40	50	60	70	80	90	100

6. Do you find it frustrating to have to consider your knee with respect to your sport?

Extremely frustrating — Not at all frustrating

☐	☐	☐	☐	☐	☐	☐	☐	☐	☐	☐
0	10	20	30	40	50	60	70	80	90	100

ACL – RSI continued

7. Are you fearful of re-injuring your knee by playing your sport?

Extremely fearful										No fear at all
☐	☐	☐	☐	☐	☐	☐	☐	☐	☐	☐
0	10	20	30	40	50	60	70	80	90	100

8. Are you confident about your knee holding up under pressure?

Not at all confident										Fully confident
☐	☐	☐	☐	☐	☐	☐	☐	☐	☐	☐
0	10	20	30	40	50	60	70	80	90	100

9. Are you afraid of accidentally injuring your knee by playing your sport?

Extremely afraid										Not at all afraid
☐	☐	☐	☐	☐	☐	☐	☐	☐	☐	☐
0	10	20	30	40	50	60	70	80	90	100

10. Do thoughts of having to go through surgery and rehabilitation prevent you from playing your sport?

All of the time										None of the time
☐	☐	☐	☐	☐	☐	☐	☐	☐	☐	☐
0	10	20	30	40	50	60	70	80	90	100

11. Are you confident about your ability to perform well at your sport?

Not at all confident										Fully confident
☐	☐	☐	☐	☐	☐	☐	☐	☐	☐	☐
0	10	20	30	40	50	60	70	80	90	100

12. Do you feel relaxed about playing your sport?

Not at all relaxed										Fully relaxed
☐	☐	☐	☐	☐	☐	☐	☐	☐	☐	☐
0	10	20	30	40	50	60	70	80	90	100

5. Things to look out for

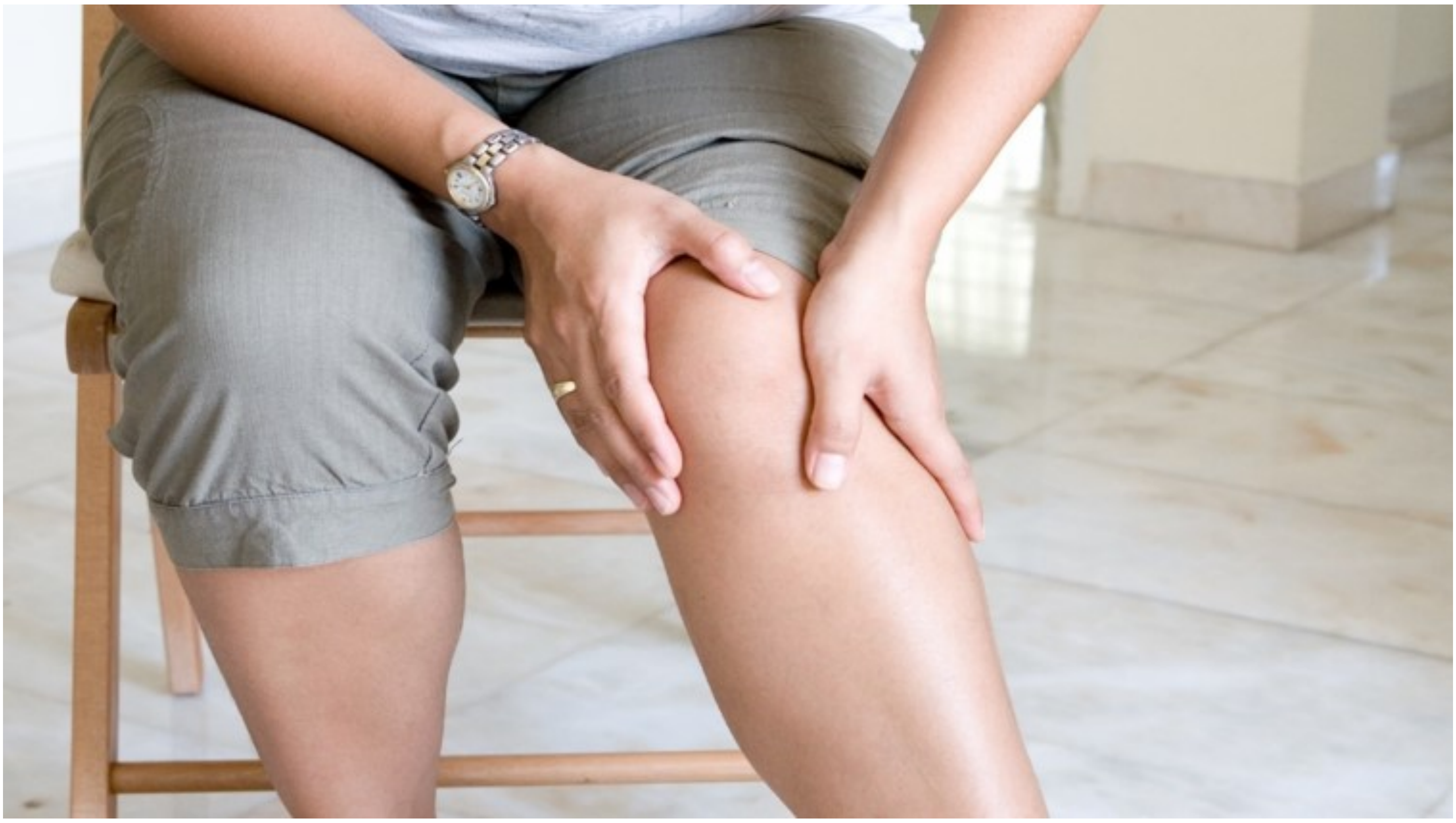

This education leaflet is designed to give you a brief and basic overview of signs and symptoms to look out for following anterior cruciate ligament reconstructive surgery

Every patient, injury and surgeon is different therefore please consult your health professional for further individual detail

Further information related to this topic can be found at:

semrc.blogs.latrobe.edu.au

Many people experience new symptoms and change in function of their knee, and it is important to know that this is normal and common after ACL reconstructive surgery.

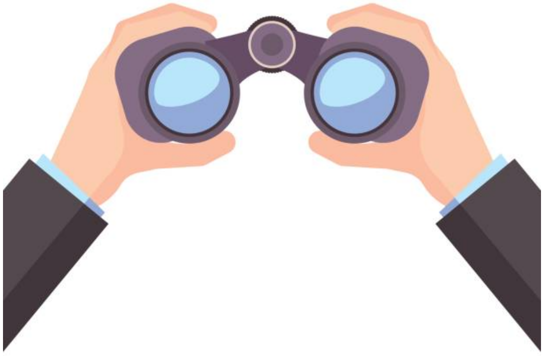

PATIENT EDUCATION
ANTERIOR CRUCIATE LIGAMENT RECONSTRUCTION

Future symptoms – am I at risk?

Some people experience changes in symptoms and function in the early years following their surgery, where as others it occurs much later and others not at all. It is poorly understood what puts people into these categories. And it is likely that a large variety of factors play a part in the development of symptoms.

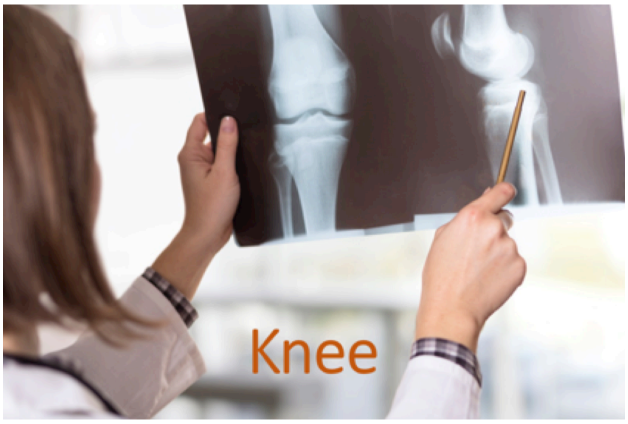

Key things to watch for

- New or change in pain, swelling or stiffness (at rest, during or after activity)
- Giving way
- People stating you are limping whilst walking
- Altered muscle strength or weakness

What is osteoarthritis? Will I get it?

Knee osteoarthritis (OA) is a common musculoskeletal condition with incidence increasing with age.

Isn't it just a normal part of ageing?
Now that you have had an ACL injury and surgery your knee is at greater risk of osteoarthritis (OA) development at an earlier age

Does surgery reduce my risk of OA?
No, it restores the stability of the knee joint but it does not reduce the risk of OA.

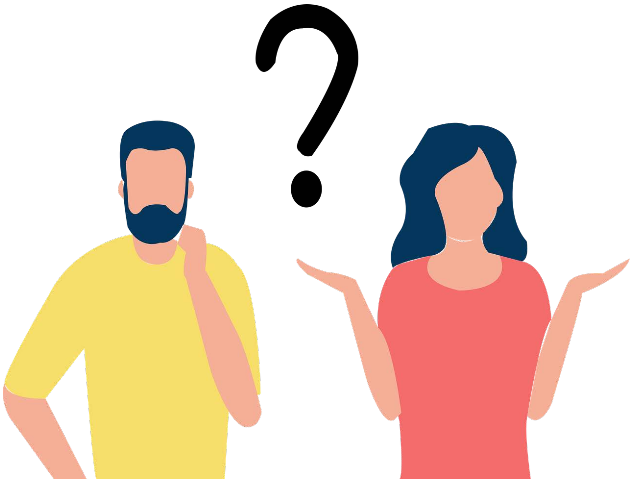

Further information

Website/Blog
semrc.blogs.latrobe.edu.au/category/acl

Facebook
facebook.com/latrobesemrc

Twitter
twitter.com/LaTrobeSEM

PATIENT EDUCATION
ANTERIOR CRUCIATE LIGAMENT RECONSTRUCTION

LA TROBE UNIVERSITY

OSTEOARTHRITIS – General Population

Knee OA (on X-ray)
1% aged between 25 - 34 years
15 – 30% aged 55 years
50% aged > 75 years

- Cartilage begins to thin
- Small bone spurs/cysts
- Space between joint becomes smaller

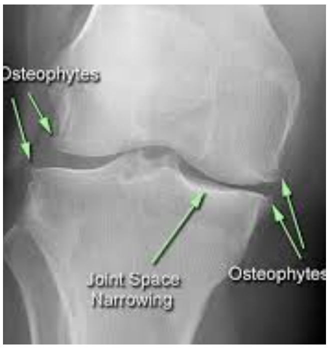

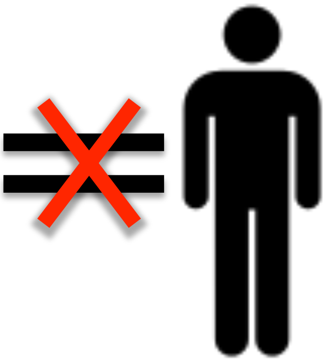

Knee pain/symptoms
<1% aged 25 - 34 years
20% aged 55 years
25% aged > 75 years

- Pain,
- Stiffness,
- Swelling,
- Loss of strength/function

THESE DO NOT ALWAYS MATCH UP
I.e. you can have degenerative changes on X-ray but NO symptoms or vice

OSTEOARTHRITIS – Following ACL Reconstruction

OA after ACL reconstruction is common and NOT always associated with symptoms

- 1 year following ACL reconstruction: 5% (I.e. aged 18 – 40)
- 15 years following ACL reconstruction: 50% (I.e. aged 35 – 60)

What can I do about it?

- **Graded exercises and return to activity** following injury is important to ensure the knee joint is gradually loaded.
- Progression based on **achieving strength/functional goals** not a time point
- Continue **seeing health care professional** until all deficits are restored
- Keep strong and maintain good function
- **Healthy diet and weight** (seek professional help if unsure)

Further information

Website/Blog
semrc.blogs.latrobe.edu.au/category/acl

Facebook
facebook.com/latrobesemrc

Twitter
twitter.com/LaTrobeSEM

- Assessment -

RETURN TO RUNNING

Prior to commencing a running program, it is best to ensure the knee is strong enough in all appropriate muscle groups, the patient has good neuromuscular control and has pre empted running with other forms of cardiovascular load

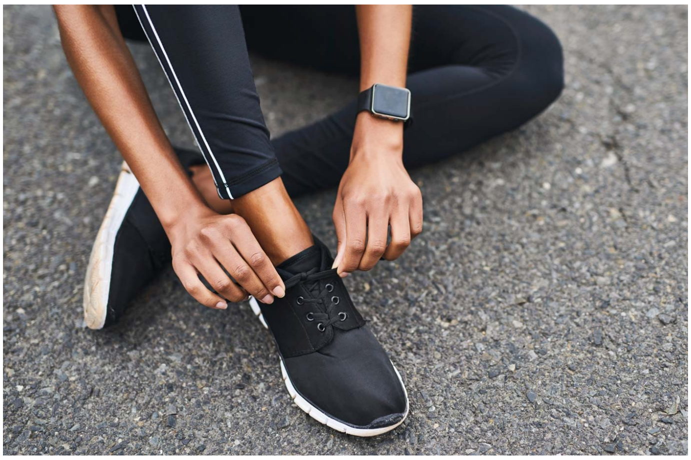

Patient Name	Pain	Swelling	Strength	Strength	Strength	Cardiovascular
	< 2/10	Zero-1+	1RM SQUAT 50-70% BW<br><br>OR<br><br>SL Leg Press 1.0 BW	> 90% LSI SL glut bridge and > 10<br><br>> 90% LSI SL calf raise and >15<br><br>> 70% LSI hamstring at 45 and 15 degrees<br><br>>90% LSI side bridge and > 30s	>22 One Leg Rise Test (modified height)<br><br>AND<br><br>Pass SL Squat functional criteria	Completes 30 minutes continuous CVS exercise<br>≥ 3 x week
**Date:**						
**Date:**						

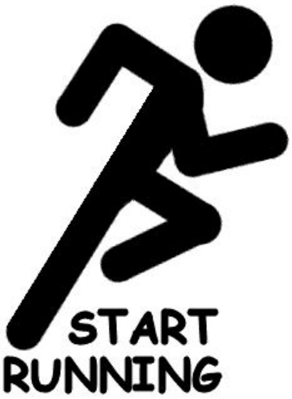

How much do I run?

The knee will best respond to gradual load. Sometimes walk – jog is recommended. As a basic rule, don't increase total load/distance by more than 15% each week.

See Section 9 – Example of Graded Running Program

Depends on your sport/goals

DATE:	MODIFIED MELBOURNE RETURN TO SPORT SCORE			
**PART A**	**Raw Score**	**Limb symmetry index % / Scoring system**		**Converted**
Effusion: Stroke test		Absent: 5 pts Present: 0 pts		**/5**
Stability: Lachman's		Nil: 5 pts Mild: 3 pts Moderate: 0 pts		**/5**
Flexion: Supine	Left: Right:	0-5°deficit: 5 pts 5-20°deficit: 3 pts, >20°: 0 pts		**/5**
Extension: Prone leg hang	Left:<br>Right:	0-2cm deficit: 5 pts 2-5cm deficit: 3 pts >5cm deficit: 0 pts		**/5**
Total Part A				**/20**
**PART B:**<br>ACL-RSI (Via App)	/120	X 100 = % Score		Divide by 5
Total Part B				**/20**
**PART C**		Otherwise score 0		
Star excursion Balance	Left: Ant: PM: PL:<br>Right: Ant: PM: PL:	LSI > 95% all three directions= 3 points	> Normative value = 2 points	**/5**
Single Hop	Left<br>Right	LSI > 95% = 3 points	> Normative value = 2 points	**/5**
Side Hop	Left<br>Right	LSI > 95% = 3 points	> Normative value = 2 points	**/5**
One Leg Rise	Left<br>Right	LSI > 95% = 3 points	> Normative value = 2 points	**/5**
Hamstring Strength	Left (SL hamstring bridge max reps)<br>Right	LSI > 95% = 3 points	> Normative value = 2 points	**/5**
Hip strength	Left (Abduction HHD)<br>Right	LSI > 95% = 3 points	> Normative value = 2 points	
Squat	BW: Squat load:<br>RM:	External load: 50% BW 6RM = 1 point,<br>75% BW 6RM = 3 points, 100% BW 6RM = 5 points		**/5**
Quality of Movement	LESS SCORE:	Excellent (0-3 points on LESS): award 5 points<br>Good (3-6 points on LESS): award 2 points<br>Poor (> 6 points on LESS): award 0 points<br>(Score 0 if landing, running or agility program not yet completed to required dosage* see program)		
	Double leg land:			**/5**
	Left leg land:			**/5**
	Right leg land:			**/5**
	Agility:			**/5**
Total Part C				**/60**
**SCORE**				**/100**

Pain

Test Description	Scoring	Goal/Pass
1. Ask the patient if they have had knee pain in the last week where is that pain.<br><br>2. Ask the patient when they experience this pain<br><br>3. Ask the patient to rate the pain /10 on VAS scale (0 no pain to 10 worst imaginable pain)	Location: AM PFJ (anterior medial patelllofemoral).<br><br>When: Ie. During running/particular exercise<br><br>Intensity: / 10 VAS scale	For any progression must have <2/10 during/after activity

Swelling

Test Description	Scoring	Goal/Pass
**Effusion**<br>*Stroke Test:*<br>Supine, knee in full extension and relaxed. Hand beneath medial tibiofemoral joint line, stroke hand upwards towards suprapatellar bursa for a 2-3 times in sweeping motion in attempt to move effusion from the inside of the joint to suprapatellar pouch. Then stroke downwards on the lateral aspect of the knee (thigh), just superior to the suprapatellar bursa towards the lateral joint line.	Zero: no wave produced on downstroke<br>Trace: small wave on medial side with downstroke<br>1+: Large bulge on medial side with downstroke<br>2+: Effusion spontaneously returns to medial side after upstroke<br>3+: So much fluid that it is not possible to move the effusion out of the medial aspect of the knee	For any progression must have: Zero – 1+

Range of Motion

Test Description	Scoring	Goal/Pass
**Flexion**<br>Patient supine slide heel up towards bottom<br>Keep heel and pelvis on ground<br>Active movement no passive help from hand or therapist	Goniometer in degrees<br>Record any pain / catching	0 – 5 deficit

**Extension**<br>Prone leg hang<br>Knees on edge of plinth but not off<br>Relax lower leg and feet<br>Measure heel height difference without placing pressure on heel<br>Use inclinometer if available	Centimetre heel height difference<br>Record ACLR or CL more flexed	< 2 cm

Stability

Test Description	Scoring (for return to sport score)	Goal/Pass
**Lachman's test**<br>Knee flexed 20-30 degrees in supine<br>One hand behind tibia and other grasping thigh<br>Thumb on tibial tuberosity<br>Use other side as comparison	Nil (laxity): 5 points<br>Mild: 3 points<br>Moderate: 0 points	NA

TASK 1: Movement Retraining

To move to next phase / type of movement retraining the following must be achieved

Test Description	Scoring	Goal/Pass
**Double Leg Landing**<br>- Double leg land LESS (Landing Error Scoring System)	Excellent = 0 – 3 points<br>Good = 4-5 points<br>Moderate = 6 points<br>Poor = < 6 points	Excellent
**Single Leg Landing (ACLR and CL)**<br>- Single leg land Modified LESS (Landing Error Scoring System)	Excellent = 0 – 3 points<br>Good = 4-5 points<br>Moderate = 6 points<br>Poor = < 6 points	Excellent
**Running**<br>- "Return to Running Criteria" see attached (pain, swelling, strength, function, graded CV program criteria)	See attached	Pass all measures
**Agility**<br>- Modified movement error scoring system	Excellent = 0 – 3 points<br>Good = 4-5 points<br>Moderate = 6 points<br>Poor = < 6 points	Excellent

DOUBLE LEG LANDING ERROR SCORING

Front view	Lateral (side) trunk flexion at contact – trunk is flexed	Yes=0, No=1
	Knee valgus angle at contact – knees over the midfoot	Yes=0, No=1
	Knee valgus displacement – knees inside of large toe	Yes=1, No=0
	Foot position at contact - toes pointing out greater than 30 degrees	Yes=1, No=0
	Foot position at contact - toes pointing out less than 30 degrees	Yes=1, No=0
	Stance width at contact - less than shoulder width	Yes=1, No=0
	Stance width at contact - greater than shoulder width	Yes=1, No= 0
	Initial foot contact – symmetric	Yes=0, No=1
Side View	Hip flexion angle at contact – hips are flexed	Yes=0, No=1
	Trunk flexion angle at contact – trunk in front of hips	Yes=0, No=1
	Knee flexion angle at contact – greater than 30 degrees	Yes=0, No=1
	Ankle plantar flexion angle at contact – toe to heel	Yes=0, No=1
	Hip flexion at max knee flexion angle – greater than at contact	Yes=0, No=1
	Trunk flexion at max knee flexion – trunk in front of the hips	Yes=0, No=1
	Knee flexion displacement – greater than 30 degrees	Yes=0, No=1
	Sagittal plane joint displacement	Large motion (soft) = 0, Average = 1, Small (loud/stiff) =2)
	Overall Impression	Excellent = 0, Average = 1, Poor = 2

SINGLE LEG LANDING ERORR SCORING

	LEFT	SCORE:
Front view	*Lateral (side) trunk flexion at contact –excessively flexed to the side*	Yes=1, No=0
	Knee valgus angle at contact – knees over the midfoot	Yes=0, No=1
	Knee valgus displacement – knees inside of large toe	Yes=1, No=0
	Foot position at contact - toes pointing out greater than 30 degrees	Yes=1, No=0
	Foot position at contact - toes pointing out less than 30 degrees	Yes=1, No=0
	Pelvic movement – lateral deviation or shunt or pelvic drop	Yes=1, No=0
	Hip is adducted	Yes=1, No=0
	Maintains balance on one leg	Yes=0, No=1
Side View	Hip flexion angle at contact – hips are flexed	Yes=0, No=1
	Trunk flexion angle at contact – trunk in front of hips	Yes=0, No=1
	Knee flexion angle at contact – greater than 30 degrees	Yes=0, No=1
	Ankle plantar flexion angle at contact – toe to heel	Yes=0, No=1
	Hip flexion at max knee flexion angle – greater than at contact	Yes=0, No=1
	Trunk flexion at max knee flexion – trunk in front of the hips	Yes=0, No=1

	Knee flexion displacement – greater than 30 degrees	Yes=0, No=1
	Sagittal plane joint displacement	Large (large/soft) = 0, Average = 1, Small (loud/stiff) =2)
	Overall Impression	Excellent = 0, Average = 1, Poor = 2

AGILITY MOVEMENT SCORING

	LEFT	SCORE:
View from front	Frontal plane foot placement > 1 foot away from CoM	Yes=1, No=0
	Upright (<45 hip knee flexion)	Yes=0, No=1
	Excessive torso lean away from direction going	Yes=1, No=0
	Foot position at contact - toes pointing out greater than 30 degrees opposite to direction going	Yes=1, No=0
	Excessive torso rotation away from direction going	Yes=1, No=0
	Overall Impression	Excellent = 0, Average = 1, Poor = 2

TASK 2: Squats

Test Description	Scoring	Goal/Pass
**Squat**<br>- When reviewing exercise program note the maximum weight patient can lift for each exercise with safety in mind, good technique, alignment and no pain.	Record in log book<br>- Load and repetitions	Return to running: 50% BW 6RM<br><br>Return to sport<br>50% BW 6RM = 1 point,<br>75% BW 6RM = 3 points<br>100% BW 6RM = 5 points
**Leg Press (if available can be used for interlimb differences)**<br>- 1RM Single Leg<br>- Perform adequate warm up at lower weights	R and L KG/pounds	Return to running: 1.0 x BW 1RM<br><br>Return to sport: 1.5 x BW 1RM

TASK 3: Balance

Test Description	Scoring	Goal/Pass
**Single Leg Stance**<br>- Arms crossed over chest<br>- Hip and knee maintained at 90 degrees<br>- Functional alignment maintained (i.e. no lateral deviation/flexion of the trunk, hips level, trunk neutral.	Max number seconds<br>Best of 3 trials	Return to running/sport: 10s eyes closed

**Star Excursion Balance Test**<br>- Shoes off, hands on hips, minimal stance foot movement allowed (ie rotation or lift)<br>- Trunk movement allowed if controlled movement<br>- Most distal toe for anterior on line, Heel for posterior directions<br>- 2 practice trials each leg, 3 good trials on each, continue until plateau of performance	Record best trial<br><br>Anterior: cm<br>Posterior medial: cm<br>Posterior lateral: cm	Return to sport:<br><br>LSI > 95% all three direction = 3 points<br><br>> Normative value for age/gender = 2 points<br><br>Otherwise score 0

TASK 4 and 6 (Hip Abductors + Trunk)

Test Description	Scoring	Goal
**Side bridge**<br>- Knees bent<br>- Shoulder, hips, knee and ankle in line<br>- Hips fully extended<br>- Continue until unable to maintain correct position	Max number seconds	Return to running/sport:<br>> 95% LSI<br>> 30s

TASK 5: Calf

Test Description	Scoring	Goal
**Calf**<br>- Single Leg Heel Raise on edge of step (knees bent and straight)<br>- Elevate as high as possible<br>- Assessor places hand above head at highest point<br>- 1 repetition every 2 seconds, to touch assessor hand<br>- Stop as soon as unable to raise to original height or slows below the cadence above.	Maximum number of reps<br>Max at 40	Return to running/sport:<br>> 95% LSI<br>> 25 reps<br><br>Both knees bent and straight

TASK 7: Gluteal/Hamstring

Test Description	Scoring	Goal
**Hamstring Option 1 – Hand held dynamometry**<br>- Patient lying prone<br>- Measure at 15 degrees KF and 45 degrees KF<br>- Hand held dynamometer 3 trials<br>-<br>**Hamstring Option 2 - Maximum single leg hamstring bridge (used for return to sport score)**<br>- Patient supine so HF 30, KF 30 foot on chair/plinth<br>- Tester to identify max height of full hip extension<br>- Place ruler across pelvis to ensure patient reaches this height with each rep<br>- X 2 practice repetitions	Record at both 15 and 45 degrees average newtons<br><br>Record number of repetitions for each leg	Return to running:<br>- > 70% LSI<br>Return to sport:<br>- 90% LSI and > normative value for age/gender/BW<br><br>*Return to sport score*<br>LSI > 95% all three direction = 3 points<br>> Normative value for age/gender = 2 points<br>Otherwise score 0
**Gluteals - Single leg Bridge**<br>- Weight bearing leg: KF 60 degrees<br>- Non weight bearing leg: full KE, thigh level with WB thigh<br>- Arms crossed over chest<br>- Elevate hips as high as possible<br>- Assessor places hands at this height<br>- Repeat as many times as possible touching assessors hands each time<br>- Stop as soon as unable to bridge to original height	Maximum number of reps<br>Max at 40	Return to running:<br>> 95% LSI<br>> 15 reps

Functional Testing

Test Description	Scoring	Goal/Pass
**One Leg Rise Test**<br>Patient sits on an adjustable plinth.<br>Foot placed so heel is 10cm from edge of bed<br>Plinth adjusted so knee is flexed to 90 degrees<br>Arms crossed across chest<br>Opposite leg suspended knee fully extended, hip at 30 degrees<br>Rise on and off bed, allowing to just touch the surface with their bottom but are not allowed to completely sit or rest<br>Completed at speed of 45bpm (MetroTimer App)	Number of reps completed<br>Test stops if;<br>- > 3 loss of balance or other foot touching ground<br>- Not keeping up with speed<br>- Patient stops due to fatigue<br>- Patient reaches 50 reps	Return to running: >22<br><br>Return to sport: >95% LSI

Test Description	Scoring	Goal
**Single Leg Hop For Distance**<br>- *Set up:* rigid tape with measuring tape (up to 150-200cm)<br>- *Start position*: toes behind line, hand together behind back, NWB leg can do anything but touch the ground. WB leg – no limitations.<br>- *Land position*: must "stick" landing. I.e. no secondary movements/hops. Must maintain balance and not touch NWB foot > 3 secs/assessor confident balance has been controlled. NWB not allowed to touch other leg. hands must remain clasped behind back<br>- *Record*: Continue on same leg until "plateau" i.e. no longer improving. Must record 3 good trials.	Maximum score (cm)<br>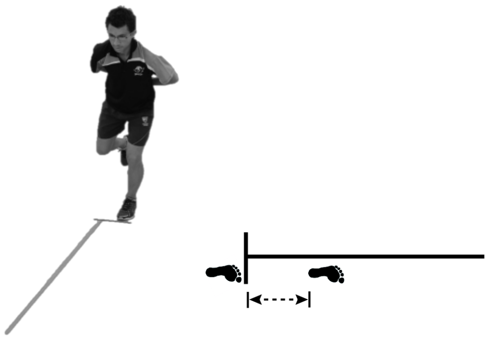	Return to running:<br>NA<br><br>Return to sport:<br>>95% LSI<br>Normative value for age/gender

Test Description	Scoring	Goal
**Side Hop**<br>- *Set up:* rigid tape 30cm apart<br>- *Start position*: toes behind line, hand together behind back, NWB leg can do anything but touch the ground. WB leg – no limitations.<br>- *Land position*: must "stick" landing. I.e. no secondary movements/hops. Must maintain balance and not touch NWB foot > 3 secs/assessor confident balance has been controlled. NWB not allowed to touch other leg. hands must remain clasped behind back<br>- *Record*: Continue on same leg until "plateau" i.e. no longer improving. Must record 3 good trials.	Maximum reps in 30s<br>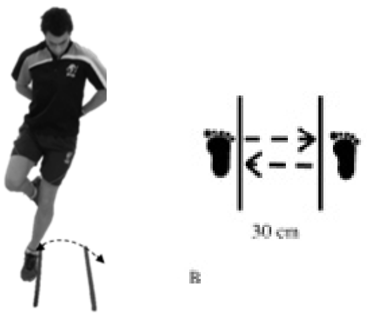	Return to running:<br>NA<br><br>Return to sport:<br>>95% LSI<br>Normative value for age/gender
**LESS Score**<br>**-** See Section 4.5 for how to score the quality of movement during double and single leg landings	Record the LESS for right and left limb on a single leg hop	0-3 points

Test Description	Scoring	Goal
**Triple Crossover Hop**<br>- *Set up:* rigid tape x 2 with measuring tape (up to 600cm), 15cm apart<br>- *Start position*: toes behind line, hand together behind back, NWB leg can do anything but touch the ground. WB leg – no limitations.<br>- *Land position*: must "stick" landing. I.e. no secondary movements/hops. Must maintain balance and not touch NWB foot > 3 secs/assessor confident balance has been controlled. NWB not allowed to touch other leg.<br>- Hands must remain clasped behind back<br>- *Record*: Continue on same leg until "plateau" i.e. no longer improving. Must record 3 good trials.	Maximum score (cm)<br>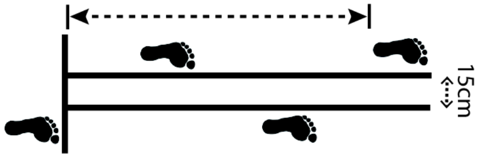	Return to running:<br>NA<br><br>Return to sport:<br>>95% LSI<br>Normative value for age/gender
**LESS Score**<br>**-** See Section 4.5 for how to score the quality of movement during agility cuts	Record the LESS for right and left limb for a 90 degree cut	0-3 points

THANK YOU

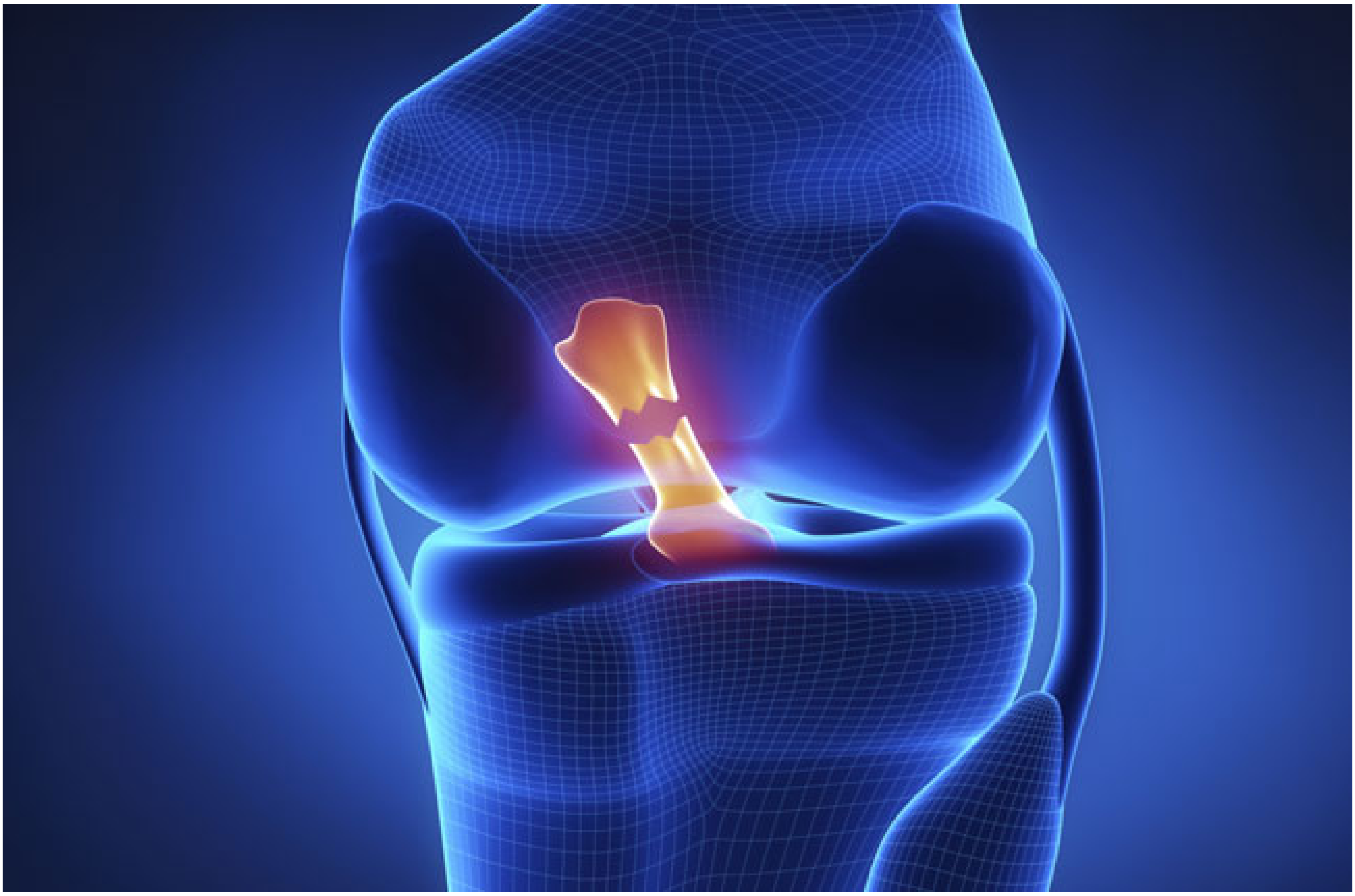

Investigato rs: Ms Brooke Patterson, Professor Kay Crossley, Dr Adam Culvenor, and Dr Christian Barton.

If you have any questions relating to the project please contact Chief Investigator – Professor Kay Crossley. This project is approved by the La Trobe Human Ethics Committee (HEC-16-077)

Please remember – investigator Brooke Patterson is blinded to patient group

LA TROBE UNIVERSITY

Sport and Exercise Medicine Research Centre
