# Supplementary material for: Exercise-therapy and education for individuals one year after anterior cruciate ligament reconstruction: a pilot randomised controlled trial
Source: BMC Musculoskelet Disord. 2021 Jan 11;22:64. doi: 10.1186/s12891-020-03919-6 (PMC7802328; doi:10.1186/s12891-020-03919-6)
Supplement: Supplementary file 3 — Additional file 3. Lower-limb focussed and trunk-focussed exercise-therapy and education interventions website https://task.trekeducation.org/. [file 12891_2020_3919_MOESM3_ESM.docx]

**Additional File 3**

Lower-limb focussed and trunk-focussed exercise-therapy and education interventions website <https://task.trekeducation.org/>
