# Supplementary material for: Exercise-therapy and education for individuals one year after anterior cruciate ligament reconstruction: a pilot randomised controlled trial
Source: BMC Musculoskelet Disord. 2021 Jan 11;22:64. doi: 10.1186/s12891-020-03919-6 (PMC7802328; doi:10.1186/s12891-020-03919-6)
Supplement: Supplementary file 4 — Additional file 4. Feedback from study participants on exercise program content, structure and delivery methods. [file 12891_2020_3919_MOESM4_ESM.pdf]

Additional File 4. Feedback from participants at follow-up

Questions each participant (n=23) was asked at follow-up

1. Were you aware which intervention group you were in? Did you think you were in the control group?
2. Do you have any feedback on the exercise program (specifically the content, and any barriers to completing the program in own time)?
3. Do you have any feedback on the exercise program delivery (specifically the physiotherapist, duration and frequency of appointments, and any barriers to attending physiotherapy)?
4. Do you have any feedback on Physiapp?
5. Do you have any feedback on the trial as whole? (i.e. would you participate again, recruitment methods)?

1. Were you aware which intervention group you were in? Did you think you were in the control group?

ID#	
1	Wasn't sure
2	My initial concern was that if I was in control group or not. But I wasn't sure.
3	No
4	No
5	No
6	No
7	No
8	No
12	No
13	No
14	No
15	No
16	I had fair idea early on I might be in the control group, however, I thought it was a valid treatment
17	No
18	No
19	No
20	I thought was in the control group / standard program against a new innovative program, but I wasn't worried what group was in
22	No
24	No
25	No, as I felt like I needed the core strength once I started
26	Didn't know didn't think about it
28	Didn't know, wasn't concerned
30	No

2. Do you have any feedback on the exercise program (specifically the content, and any barriers to completing the program in own time)

ID#	
1	The exercises were good additions to current program, sometimes I would do my own program sometimes I would do the trial exercises
2	-
3	Too much, especially every day, not striving for elite sport, exercise 3 x week running in off days. Hard to fit it in
4	All got too much, life and work and study and travel
5	I felt benefit of core strength for work (standing all day bar work)
6	Difficult to find motivation as didn't feel was assisting knee, but overall gradual build into hockey confidence built and knee coping better and better
7	Too many exercises time constraints
8	It was good
12	Great to see progression in exercises and have consistent physiotherapy monitoring at this time point otherwise would have just continued and done nothing
13	Type of exercise needs to take into account activities that doing throughout the day. I am squatting all day at work,
14	Wasn't sure exercises were going to help knee
15	Good, challenging progressing all the time, 1 rep max squat improvement 105-135 very happy with that
16	Felt was improving deficiencies in core strength, which helped with LBP, golf swing, standing for longer periods at work, and tightness through hip flexors, good workout, good variety of different muscles and positions
17	Felt personalised and modified to make challenging for me, core strength useful for my sport/throwing athletics,
18	Good exercises
19	Didn't notice much improvement but wasn't working hard on the exercises
20	I struggled with motivation at the end as I was doing the same exercises, bored, felt wasn't progressing, as wasn't able to move forward before reaching a certain weight. Felt a bit limited by being in a trial that exercises couldn't be individualised for me
22	Felt was catered to me and specific to ACL
24	Good to begin with then felt bit boring as same exercise just increased weight
25	Really good exercises have taken photo of them all to continue doing them
26	Loved variety of exercises, but it was time consuming, I hated the bridges, favourite exercise was jumping and hopping
28	Good variety and always challenging
30	Good amount of exercises 1-hour sessions, good how was progressing each week initially, felt good to squat and increase the weight to almost body weight and see tangible improvements, would be better if there was a home option for every exercise as only couple needed gym could do most at home didn't seem worth it, bit boring in the end same exercises just more weight would like variety.

3. **Do you have any feedback on the exercise program delivery (specifically the physiotherapist, duration and frequency of appointments, and any barriers to attending physiotherapy)?**

ID#	
1	physio good at giving demonstrations
2	Dave was excellent
3	physio really good in terms of explanation, need time to be able to fully understand what meant to do at home or more regular check-ups as forget and couldn't interpret exercise program
4	Dave was excellent but overall got too much and wasn't priority at the time due to work, study and travel commitments
5	difficulty with doing exercises and attending appointments due to newborn, work, lacking motivation to return to sport
6	nil
7	good explanation of why core was needed
8	some of the instructions on video hard to understand can't remember which ones
12	good
13	good demo and explanations
14	nil
15	structure and timing no issue, 30 mins enough, 1 week initailly good, 2-3 weeks then suitable,
16	Dave was great, explaining reasons for exercises and simple instructions,
17	structure was good, enough time,
18	good spacing of appointments, more the better,
19	3 weeks too long, need motivation, 30 mins good, difficulty with missing school and work to make appointments
20	30-40 mins was enough time to go through everything,
22	30 mins enough time, structure good able to self-progress,
24	Dave was great, worked really well to update program on app if it was just an increase in weight
25	good amount of time and constantly challenged
26	Dave very accommodating, right amount of appointments once a week for any longer would be too much and inconvenient, 30 mins enough time
28	good structure of appointments, enough time
30	3-4 months good, feel like need to learn how to self-manage now with guidance as required, 6 days a week exercises too much . Will struggle once go back to work, spacing of appts good. ? Not as rigid for 3-week follow ups if need to come in for a question

4. Do you have any feedback on Physiapp©?

ID#	
1	too hard and clunky
2	good for accountability, useful for exercise technique
3	little bit clunky, not updating all the time, like the pain and additional comments section, number of sets and reps confusing and to do each side or not, videos really good, bit too wordy descriptions,
4	too much to have to fill out all the time
5	remembering to fill out an issue
6	nil
7	good, easy to use
8	easy to use, kept accountable
12	good easy to use intuitive, great to keep track of progression, unable to change comments section
13	clunky, not updating have to log in and out again videos good but music irritating, technique good vs bad really good
14	good
15	good at times, frustrating at times , especially if got a new program / program ended. Videos and instructions good for new exercises
16	bit clunky didn't always load and update, also hard to go back and enter retrospectively, videos good reminder for new exercises
17	Couldn't go back and enter in completion, would like to enter 1 x week, didn't always have phone, videos and text useful to make individualised comments
18	useful videos and instructions
19	instructions helpful , clear reps and sets , no technical issues
20	Really liked the app, no issues, issue with changing days exercising on annoying, something good would be an idea of how long they should take, best order of exercises, amount of rest time
22	Couldn't go back more than 5 days to enter in, used the text more than the videos, good to track progress and help with motivation, individual notes good
24	great, used text rather than videos as reception not great and videos used battery, no issues with filling in compliance, ability to have order of exercises would be good as figured out was better to do gluteal stuff first before squats
25	used paper logbook, liked this, would just take photo of it and then fill it in later.
26	trouble with writing comments on specific exercises , video and written instructions very good used both, clear on reps and sets, sometimes too much info / contradicting what Physio was saying on the written instructions
28	Some difficulty at times with new program loading and going back to enter in completion of exercises
30	Great! Loved the app for self-monitoring and motivation. Issue with messaging not directly linking to the exercise referring to. Would be great to be able to continue using independently.

5. **Do you have any feedback on trial as a whole (i.e. would you participate again, recruitment methods)?**

ID#	
1	overall happy with program however didn't feel like it helped with pain or range of motion, improved strength, still lacking confidence to go bush walking
2	Excellent result improvement in all measures, would participate again, going to miss not having the accountability
3	expectations should be set by surgeon - so much more to it than what they say, overall, I felt like I got stronger, but sickness other injuries (sesamoiditis) got in way of making more progress and doing exercises. I had a much better physiotherapy experience in terms of treating whole body not just the knee.
4	Nil
5	Nil
6	Nil
7	overall happy with program felt was beneficial. Function likely improved from increased basketball 1 - 4 x week and gradual loading back into basketball pain settling to manageable level
8	overall was doing quite well until strained hamstring in basketball 6 weeks ago. Found that my body awareness was quite poor I found it difficult to be able to correct techniques very well
12	overall good improvement in measures, very happy with program and progress, plans to continue to increase running and maintain gym program and see PT as required
13	overall felt good improvement in feeling of the knee and strength, hope to continue doing the program, will likely come back and see Nick for clearance for RTS, pes anserine pain flare up
14	nil
15	
16	overall good to be a part of
17	overall felt improvements and increased confidence in knee with time. Core strength helped athletics, going to netball tryouts on Monday
18	overall felt good improvement in strength and reduced amount of "instability" and effusions, will continue to do strengthening exercises, some pain with exercises but would settle back to norm
19	nil
20	overall noticed significant decrease in pain, increase in strength and really enjoyed being a part of the trial
22	overall very glad participated, overall improvement in pain and strength
24	overall felt really improved confidence and ability to do full moves at cheerleading and noticeable decrease in pain
25	overall have learnt that needs to me that is ready not surgeon saying graft is okay
26	would like to return to tennis and skiing, get into some kind of sport for competitive needs, start yoga/Pilates
28	aiming to go to world champs athletics
30	overall very happy with improvements in pain everyday activities, would like to be able to increase running tolerance and confidence in skiing. Worried about how will go without the motivation of the trial, the app, the physio
