# Supplementary material for: Exercise-therapy and education for individuals one year after anterior cruciate ligament reconstruction: a pilot randomised controlled trial
Source: BMC Musculoskelet Disord. 2021 Jan 11;22:64. doi: 10.1186/s12891-020-03919-6 (PMC7802328; doi:10.1186/s12891-020-03919-6)
Supplement: Supplementary file 5 — Additional file 5. Secondary outcomes additional detail. [file 12891_2020_3919_MOESM5_ESM.pdf]

Additional File 5, Secondary outcomes additional detail

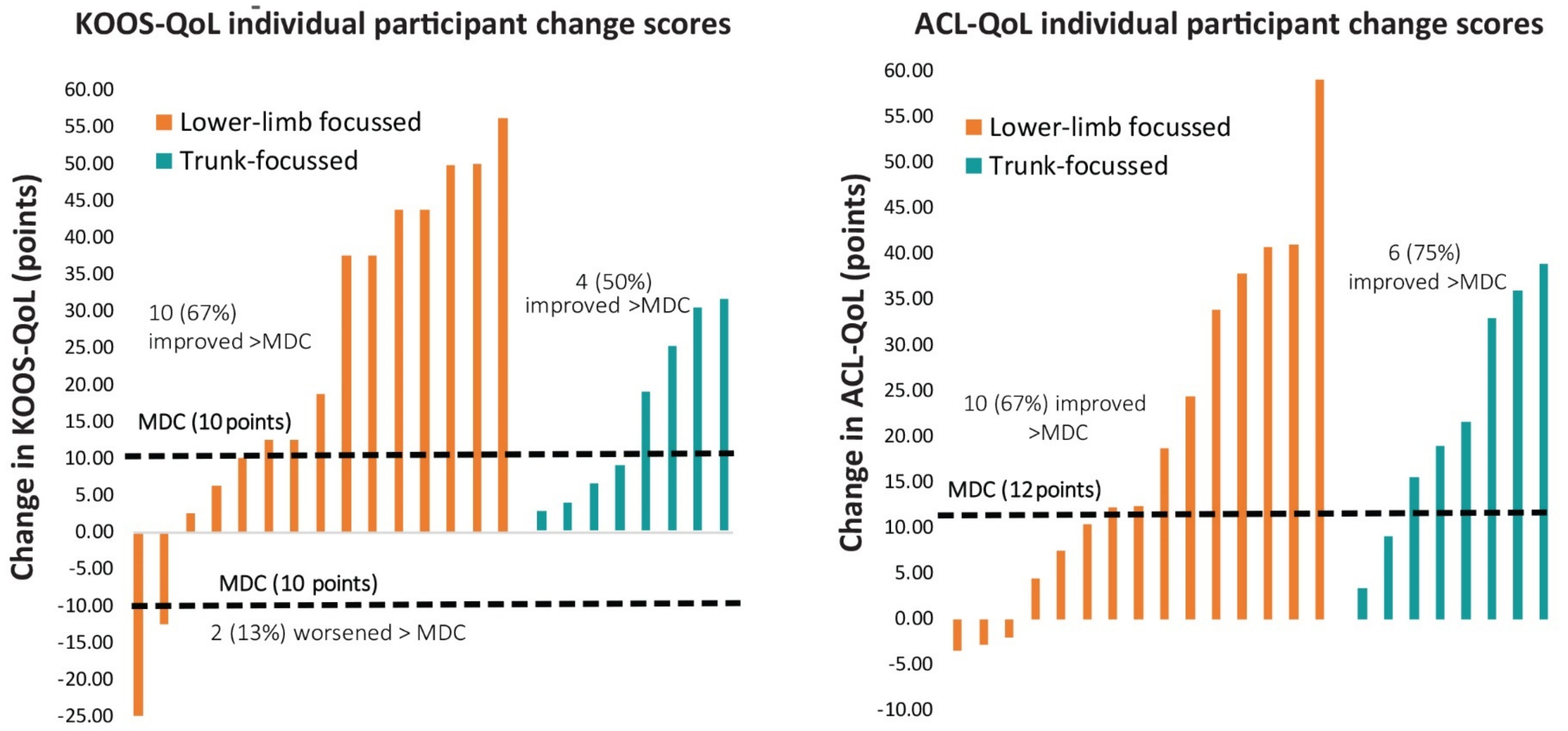

Figure 1. Individual participant changes for the KOOS-QoL and ACL-QOL

KOOS=Knee injury and Osteoarthritis Outcome Score; ACL-QoL=Anterior Cruciate Ligament Quality of Life survey; MDC=minimal detectable change for KOOS-QoL (10 points) and ACL-QOL (12 points).

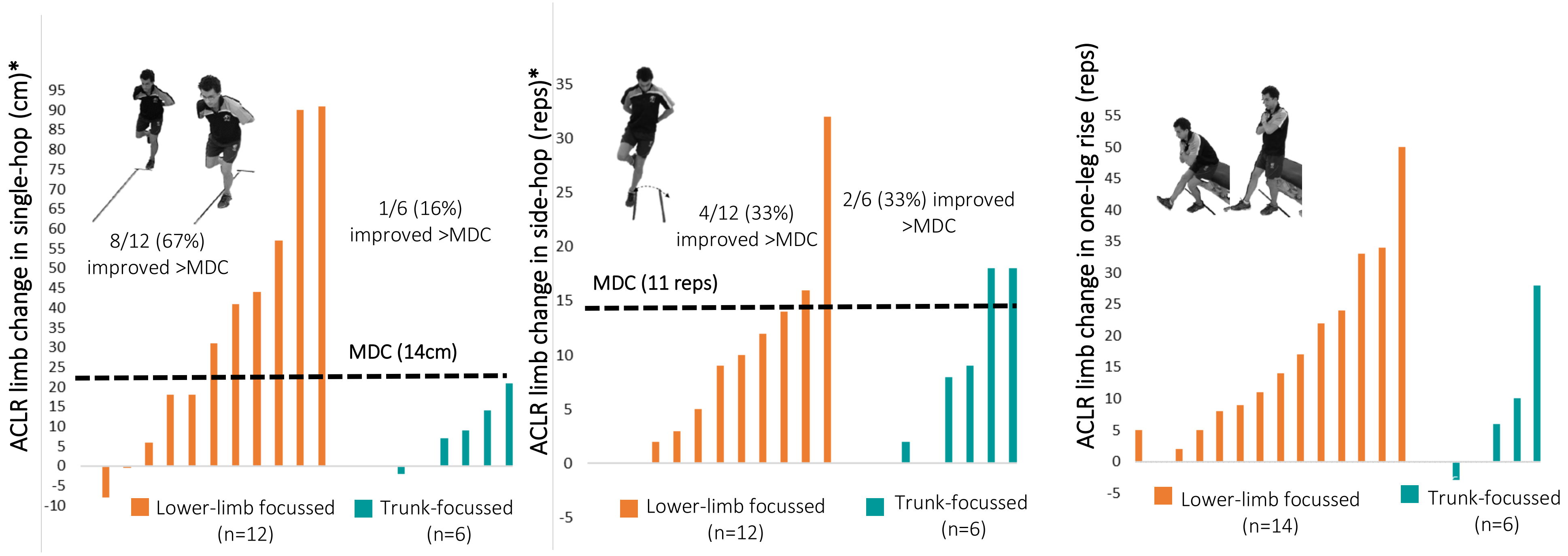

Figure 2. Individual participant changes for the functional performance in the ACLR limb

ACLR=anterior cruciate ligament reconstructed limb; MDC = minimal detectable change for the single-hop (14 cm) and side-hop (11 repetitions) from Kockum et al., (2015). No known MDC for the one-leg rise.
~n=20 out 23 completed follow-up functional performance assessment (n=2 overseas, n=1 work commitments).
* single-hop and side-hop, n=18 (n=2 could not complete the hop-tests at follow-up as they were still recovering from adverse events; n=1 hamstring strain, n=1 ankle sprain).
